# XAI-BT-EdgeNet: explainable edge-aware deep learning with squeeze-and-excitation for brain tumor detection and prediction

**DOI:** 10.3389/frai.2025.1676524

**Published:** 2025-11-20

**Authors:** Deependra Rastogi, Prashant Johri, Massimo Donelli, Tarun Agarwal, Shrikant Tiwari, Pushpa Singh

**Affiliations:** 1 School of Computer Science and Engineering, IILM University, Greater Noida, India; 2 School of Computer Science and Engineering, Galgotias University, Greater Noida, India; 3 Department of Civil, Environmental, Mechanical Engineering, University of Trento, Trento, Italy; 4 Radiomics Laboratory, Department of Economy and Management, University of Trento, Trento, Italy; 5 Department of Computer Science and Engineering, JIIT, Noida, India

**Keywords:** brain tumor, magnetic resonance imaging, deep learning, edge aware, squeeze and excitation, explainable interpretability

## Abstract

**Introduction:**

Accurate and early detection of brain tumors is critical for effective treatment and improved patient outcomes, yet manual radiological analysis remains time-consuming, subjective, and error-prone. To address these challenges and improve clinical trust in AI systems, this study presents XAI-BT-EdgeNet, an explainable, edge-aware deep learning framework integrated with squeeze-and-excitation (SE) modules for brain tumor detection using MRI scans.

**Methods:**

The proposed architecture employs a dual-branch design that fuses high-level semantic features from InceptionV3 with low-level edge representations via an Edge Feature Block, while SE modules adaptively recalibrate feature importance to enhance diagnostic accuracy. To ensure transparency, the model incorporates four XAI techniques—LIME, Grad-CAM, Grad-CAM++, and Vanilla Saliency—which provide interpretable visual justifications for predictions. The framework was trained and evaluated on the Brain Tumor Dataset by Preet Viradiya, comprising 4,589 labeled MRI images divided into Brain Tumor (2,513) and Healthy (2,076) classes.

**Results:**

The model achieved 99.58% training accuracy, 99.71% validation accuracy, and 100.00% testing accuracy, alongside minimal loss values of 0.0103, 0.0051, and 0.0026, respectively. These results demonstrate the robustness and precision of the proposed framework in brain tumor classification.

**Discussion:**

This work includes the development of a dual-branch CNN architecture that combines semantic and edge features for enhanced classification, the integration of SE modules to highlight clinically significant regions, and the application of multi-method XAI to offer transparent, interpretable outputs for clinical applicability. Overall, XAI-BT-EdgeNet delivers a high-performing, interpretable solution that bridges the gap between deep learning and trustworthy clinical decision-making in brain tumor diagnosis.

## 1 Introduction

Brain tumors are among the most complex and fatal diseases affecting the central nervous system, with substantial implications for neurological function, cognitive performance, and overall quality of life (Chieffo et al., 2023; Pancaldi et al., 2023). Globally, brain tumors contribute to a significant proportion of cancer-related deaths, especially among children and older adults. Their biological heterogeneity and unpredictable growth patterns pose serious diagnostic and therapeutic challenges. Accurate and early diagnosis is paramount to improving treatment planning and patient outcomes, as delayed detection often leads to rapid disease progression and reduced survival rates (National Institutes of Health, 2025).

Magnetic resonance imaging (MRI) is the primary imaging modality used for non-invasive visualization of brain tumors due to its ability to capture high-resolution anatomical detail and tissue contrast without ionizing radiation (Peng et al., 2025; Misra, 2024). However, interpreting MRI scans is inherently complex and highly dependent on radiologists expertise. Manual evaluation is often subjective, time-intensive, and prone to intra- and inter-observer variability. In resource-constrained healthcare environments or high-volume settings, the reliance on manual interpretation can result in diagnostic delays or oversight of subtle pathological features (Grover et al., 2015). These limitations have prompted the development of automated decision-support systems to assist in tumor detection and classification.

Deep learning, particularly convolutional neural networks (CNNs), has revolutionized the field of medical image analysis, offering robust capabilities in learning hierarchical feature representations from imaging data (Mienye et al., 2025; Mall et al., 2023; Gupta et al., 2021). CNN-based models have demonstrated excellent performance in various brain tumor classification tasks, owing to their ability to automatically extract both low-level and high-level image features (Thakur et al., 2024; Alzubaidi et al., 2021). However, a critical barrier to the clinical adoption of these models lies in their “black-box” nature. Most CNNs provide predictions without any accompanying rationale, making it difficult for clinicians to trust or interpret the decision-making process. This opacity is particularly concerning in high-stakes environments such as oncology, where diagnostic precision and accountability are essential.

Adding to the complexity of brain tumor classification is the need to distinguish not only between tumor types but also tumor grades. According to the World Health Organization (WHO), brain tumors are classified into four grades (I to IV) based on histological features such as cellularity, mitotic activity, microvascular proliferation, and necrosis (Osborn et al., 2022; Louis et al., 2016).

- Grade I tumors are typically benign, slow-growing, and often curable through surgical resection (e.g., pilocytic astrocytoma).
- Grade II tumors are low-grade malignancies with a potential to recur or progress.
- Grade III tumors exhibit more aggressive growth and cellular atypia, while
- Grade IV tumors, such as glioblastoma multiforme (GBM), are highly malignant with poor prognosis and high recurrence rates.

The ability to accurately classify both tumor type and grade from MRI scans is critical for determining prognosis and guiding treatment strategies, but remains a difficult task even for experienced radiologists (Farahani et al., 2022). Misclassification can lead to inappropriate treatment protocols and adverse patient outcomes. Furthermore, while deep learning models excel at extracting semantic features—patterns that describe the broader visual context—they often underutilize critical edge and boundary information. In the case of brain tumors, the shape, margin clarity, and texture around lesion boundaries carry valuable diagnostic clues. Tumors of higher grades often exhibit irregular, infiltrative, or necrotic edges, which may not be captured effectively by standard convolutional architectures focused solely on semantic abstraction (Kunimatsu et al., 2021). This highlights the importance of integrating edge-aware mechanisms into deep learning pipelines for enhanced diagnostic fidelity. To overcome these challenges, this study introduces XAI-BT-EdgeNet, an explainable deep learning architecture tailored for the detection and grading of brain tumors from MRI images. The proposed model features a dual-branch architecture: one branch is based on the InceptionV3 network to capture semantic-level information, while the other is an Edge Feature Block that processes gradient-based edge representations to enhance structural detail. The fusion of these complementary branches allows the model to learn both high-level features and fine-grained edge cues critical for accurate tumor classification.

To further refine the representational power of the network, squeeze-and-excitation (SE) blocks are incorporated to dynamically recalibrate channel-wise feature responses, ensuring the network focuses on diagnostically salient regions. Moreover, to ensure transparency and foster clinical trust, the framework is intrinsically designed with a suite of Explainable Artificial Intelligence (XAI) tools, including Grad-CAM, Grad-CAM++, LIME, and Vanilla Saliency. These modules produce visual explanations that allow clinicians to understand and validate the regions influencing each prediction, whether tumor type or grade.

The proposed study is guided by the following key contributions:

- Edge-aware dual-branch architecture: A dual-branch framework combining InceptionV3 with an Edge Feature Block to capture both semantic and boundary-level features, improving brain tumor detection and grading.
- Feature enhancement with SE modules: Integration of squeeze-and-excitation blocks to adaptively emphasize important features, enhancing classification accuracy across tumor types and grades.
- Built-in explainability with multi-XAI support: Use of Grad-CAM, Grad-CAM++, LIME, and Vanilla Saliency to generate interpretable visual explanations, promoting transparency and clinical trust.

Following these contributions, the remainder of this study is structured as follows: Section 2 provides a detailed literature review, highlighting existing methods in brain tumor detection and their limitations in terms of interpretability and feature representation. Section 3 outlines the dataset preparation, pre-processing pipeline, and the adapted dual-branch architecture integrated with squeeze-and-excitation modules. Section 4 discusses the evaluation criteria using standard performance metrics. Section 5 presents the experimental results, including classification performance and interpretability analysis using multiple XAI methods such as LIME, Grad-CAM, Grad-CAM++, and Vanilla Saliency. Section 6 offers an in-depth discussion of the findings, addressing the model’s strengths, practical implications, and limitations. Finally, Section 7 concludes the study by summarizing the key outcomes and suggesting directions for future research.

## 2 Literature review

Alzahrani (2023) introduced ConvAttenMixer, a deep learning model for brain tumor detection and classification. This architecture combines convolutional mixers with both external and self-attention mechanisms, enabling the model to capture both local and global features effectively. The study demonstrates that ConvAttenMixer outperforms traditional CNN-based models in detecting and classifying brain tumor types using MRI scans. The integration of attention modules significantly enhances feature representation, leading to improved performance in medical image analysis.

Rasheed et al. (2023) proposed a brain tumor classification framework that combines image enhancement techniques with convolutional neural networks (CNNs) to improve diagnostic accuracy from MRI scans. The image preprocessing stage enhances critical features in MRI images, making them more distinguishable for the CNN model. The study demonstrates that this hybrid approach significantly improves classification performance across different tumor types, highlighting the effectiveness of enhanced imaging in supporting deep learning-based medical diagnosis.

AlTahhan et al. (2023) developed a refined automatic brain tumor classification system leveraging hybrid convolutional neural networks (CNNs) applied to MRI scans. The proposed method integrates multiple CNN architectures to extract diverse and complementary features, enhancing the accuracy and robustness of tumor classification. Experimental results show that this hybrid approach outperforms standard CNN models, offering a reliable solution for automated medical diagnostics in brain tumor analysis.

Özkaraca et al. (2023) proposed a deep learning-based method for classifying multiple types of brain tumors using dense convolutional neural networks (Dense CNNs) on MRI data. The Dense CNN architecture enhances feature propagation by connecting each layer to every other layer in a feed-forward fashion, which reduces the risk of vanishing gradients and encourages feature reuse. The study tested its model on publicly available MRI datasets and reported high accuracy and generalization, particularly for glioma, meningioma, and pituitary tumors. The model demonstrated robust performance even with limited data, making it suitable for real-world clinical applications.

Peng and Liao (2023) presented a deep learning classification model focused on MRI-based brain tumor diagnosis. Presented at the IEEE ECBIOS conference, their approach integrates pre-processing steps to standardize MRI data, followed by a CNN-based classification pipeline. Their system demonstrated solid performance in identifying various tumor types, with emphasis on minimizing false positives. The study’s contribution lies in its simplicity and applicability, targeting real-time diagnostic support tools for medical professionals.

Gómez-Guzmán et al. (2023) applied CNNs to classify brain tumors from MRI images, focusing on improving detection accuracy by optimizing CNN architectures (such as layer depth and filter size). They used image augmentation to improve dataset diversity and reduce overfitting. The model showed promising classification accuracy, especially in differentiating gliomas, meningiomas, and pituitary tumors. Their findings reinforce CNNs’ effectiveness in extracting spatial and contextual features from complex MRI datasets, contributing to precision in non-invasive diagnostics.

Shanjida et al. (2022) introduced a hybrid model combining CNN and K-nearest neighbors (CNN-KNN) for detecting and classifying brain tumors from MRI scans. The CNN was used to extract high-level spatial features, which were then fed into the KNN classifier for final prediction. The study emphasized model simplicity and low computational cost, making it well-suited for environments with limited processing resources. Despite its hybrid nature, the model maintained competitive accuracy and robustness, showing particular strength in handling small and imbalanced datasets.

Mercaldo et al. (2023) addressed the critical need for interpretability in AI-driven medical diagnostics by designing a CNN-based system that detects and localizes brain tumors while offering explainable outputs. The model was trained on publicly available MRI datasets and designed with a focus on maintaining high performance without sacrificing transparency. To achieve explainability, the authors integrated Grad-CAM (Gradient-weighted Class Activation Mapping), allowing visual heatmaps that highlight the specific regions influencing the classification. This is essential for clinical settings where black-box models are typically mistrusted. The CNN architecture itself followed a moderately deep structure with batch normalization and dropout layers to prevent overfitting. Performance metrics such as accuracy, precision, recall, and F1-score were above 90% across most tumor classes. The study’s main contribution lies in its human-AI collaboration approach, offering both performance and interpretability.

Gaur et al. (2022) proposed a deep learning system specifically designed to predict tumor malignancy (benign vs. malignant) using MRI data, with an added emphasis on explanation generation. They applied a custom CNN architecture, followed by the use of SHAP (SHapley Additive exPlanations) and Layer-wise Relevance Propagation (LRP) to interpret how the model arrived at each decision. The model was trained on a curated dataset, with preprocessing steps including histogram equalization and skull stripping to improve contrast and reduce irrelevant features. The CNN was fine-tuned using cross-validation, and the model achieved over 94% accuracy for binary classification. The study is notable for embedding explanation as a core component, rather than an afterthought. These insights not only improved model validation but also provided medical experts with confidence in decision boundaries.

Ahmed et al. (2023) employed a transfer learning strategy by fine-tuning VGG-16, a well-known deep CNN architecture, to classify brain tumors into multiple types (glioma, meningioma, and pituitary). They further applied explainable AI (XAI) techniques like LIME (Local Interpretable Model-agnostic Explanations) and heatmaps to visualize model reasoning. Their dataset included both axial and coronal views of MRI scans, augmented using techniques like rotation and scaling. This diversity improved generalization and helped combat overfitting. The VGG-16 model, with minimal architectural modification, achieved an accuracy of ~96% and strong class-wise precision. The integration of VGG-16 with XAI tools proved useful for highlighting lesion zones and verifying model focus. The study bridges pretrained model power with real-world interpretability, making it practical for clinical deployment.

Naseer et al. (2021) investigated the role of data augmentation in improving CNN performance for brain tumor detection. Recognizing the limitations of small medical datasets, they implemented aggressive augmentation techniques (e.g., rotation, flipping, zooming, intensity variation) to generate a robust training set. Their custom CNN, composed of multiple convolutional, pooling, and fully connected layers, was evaluated on both original and augmented datasets. The augmented pipeline yielded a 10–12% increase in accuracy, with the final model reaching ~93% accuracy. This study highlighted the importance of dataset diversity, particularly for deep learning applications in healthcare, where acquiring labeled medical data is difficult. It also showed that simple CNNs can compete with deeper architectures if trained with enriched datasets.

Bashkandi et al. (2022) proposed a novel hybrid optimization strategy combining two nature-inspired algorithms—Political Optimizer (PO) and Particle Swarm Optimizer (PSO)—to enhance the training and hyperparameter tuning of a CNN for brain tumor classification. The optimizers were applied to adjust weights, biases, and learning parameters of the CNN, which included several convolutional and max-pooling layers. The CNN was evaluated using cross-validation on a publicly available MRI dataset, and the optimizer-driven approach significantly outperformed standard gradient descent methods. Their model achieved over 97% accuracy, and the optimization process led to better convergence and fewer training epochs. The study represents a novel contribution by integrating evolutionary strategies with deep learning, allowing fine-tuned control of network behavior and avoiding local minima.

Shawon et al. (2025) tackled the dual challenge of class imbalance and model explainability in brain tumor classification. They designed a cost-sensitive deep neural network (DNN) that penalizes misclassification of underrepresented classes (like rare tumor types), helping to reduce bias toward majority classes. In addition, they incorporated explainable AI techniques (such as Grad-CAM and SHAP) to generate heatmaps for transparency. The model was trained on a real-world, imbalanced MRI dataset, showing significantly improved recall and F1-score for minority classes. This approach is vital for clinical scenarios where rare but deadly tumors must not be overlooked.

Verma and Singh (2022) proposed and compared multiple custom deep learning frameworks for classifying brain tumors using MRI data. The study covered CNNs, RNNs, and hybrid models, with a thorough evaluation on multiple datasets. Their work analyzed trade-offs in model complexity, training time, and classification performance, concluding that CNN-based frameworks with residual connections and attention blocks offered the best balance between accuracy and computational efficiency. They also implemented ensemble learning to further enhance generalization.

Kumar et al. (2021) developed a ResNet-based CNN architecture enhanced with Global Average Pooling (GAP) for multi-class classification of brain tumors. The use of residual connections helped mitigate the vanishing gradient problem, while GAP layers reduced overfitting by minimizing model parameters. The model was tested on a balanced dataset with three tumor classes and achieved an accuracy above 95%, with fast convergence and high interpretability. This method is computationally efficient, making it suitable for integration into real-time diagnostic tools.

This study (Alhassan and Zainon, 2021) introduced a modified activation function: Hard Swish-based ReLU, incorporated into a CNN for classifying brain tumors. The new activation aimed to balance the non-linearity of ReLU with the smoothness of Swish, enhancing convergence and gradient flow. Their CNN model showed improved accuracy and faster training compared to the standard ReLU-based networks. Evaluated on MRI datasets, it achieved classification accuracy around 96%, with noticeable gains in precision and recall, especially on complex tumor boundaries.

Hosny et al. (2025) introduced an ensemble learning framework that combines several CNN-based classifiers, each trained with different architectural or hyperparameter configurations. This ensemble was enhanced with explainable AI tools like Grad-CAM, giving radiologists insight into the decision-making process. The ensemble significantly outperformed individual models, achieving an accuracy of ~98% and superior generalization across tumor types. The visual explanations also validated the model’s focus on clinically relevant tumor areas, increasing its practical utility.

This study (Athisayamani et al., 2023) employed ResNet-152, a very deep CNN, for high-level feature extraction, followed by optimized dimensionality reduction techniques (e.g., PCA and LDA) to improve classifier performance and reduce computational burden. The extracted features were fed into conventional classifiers (like SVM and k-NN), showing that the hybrid deep feature + shallow classifier approach can achieve performance comparable to end-to-end deep networks, especially when computational resources are limited.

Malla et al. (2023) proposed a deep CNN integrated with Global Average Pooling (GAP) for end-to-end classification of brain tumors from MRI images. GAP eliminated the need for fully connected layers, thus reducing overfitting and improving interpretability. Their model was trained on a multi-class dataset and reached an accuracy of ~95%, performing particularly well on noisy and artifact-prone images. The authors emphasized the model’s computational efficiency and its adaptability to embedded systems or mobile devices for telemedicine.

Drawing upon the insights from the reviewed literature and aligning with the proposed research title, the following well-defined objectives have been formulated:

- Objective 1: To design an edge-aware deep convolutional neural network integrated with squeeze-and-excitation (SE) blocks for accurate brain tumor segmentation and classification.
- Objective 2: To integrate explainable artificial intelligence (XAI) methods into the proposed model to enhance the interpretability and transparency of tumor detection and classification outcomes.
- Objective 3: To benchmark the proposed XAI-BT-EdgeNet against state-of-the-art CNN, attention-based, and ensemble models in terms of classification accuracy, computational efficiency, and explainability.

Table 1 shows the related work analysis for other state-of-the-art with a research gap.

**Table 1:** Related work for other state of art model.

References	Model type	Innovation	Dataset challenge	Notable strength	Research gap
Alzahrani (2023)	ConvMixer + Attention	Combines convolutional mixers with both external and self-attention modules to capture local and global features simultaneously	Multi-class classification	Captures global–local features	Lacks interpretability and explainability in clinical decision-making
Rasheed et al. (2023)	CNN with image enhancement	Introduces a pre-processing pipeline to enhance contrast and highlight tumor regions before CNN classification	Noise in MRI	Improved feature clarity	Does not incorporate attention mechanisms or explainable AI frameworks
AlTahhan et al. (2023)	Hybrid CNNs	Integrates multiple CNN architectures to harness complementary feature extraction capabilities	MRI variability	Diverse feature extraction	Does not offer localization or interpretability of decisions
Özkaraca et al. (2023)	Dense CNN	Utilizes dense connections to maximize feature reuse and gradient flow during training	Multi-class tumors	Efficient feature reuse	Absence of explainable components for clinical transparency
Peng and Liao (2023)	CNN-24 layers	Implements a traditional CNN architecture for fast and accessible tumor classification	General classification	Feasible for deployment	Lacks architectural novelty and performance tuning
Gómez-Guzmán et al. (2023)	InceptionV3	Optimized configuration of CNN layers tailored to MRI characteristics for classification	MRI noise/artifacts	Strong baseline	Lacks integration with advanced modules like attention or hybrid models
Shanjida et al. (2022)	CNN-KNN	Employs a hybrid classifier combining deep features with k-nearest neighbors for final decision-making	Limited data	Simple and interpretable	Struggles to scale with larger and more complex datasets
Mercaldo et al. (2023)	Resnet50	Incorporates explainable AI via Grad-CAM to visualize areas of interest in MRI scans	Localization needed	Visual interpretability	Slightly higher computational demand; lacks performance optimization
Gaur et al. (2022)	CNN with dual-input	Employs explainability methods (SHAP, LRP) to interpret model outputs and provide confidence in decisions	Binary classification	Transparent model	Only binary classification addressed; not evaluated for multi-class problems
Ahmed et al. (2023)	VGG16	Combines transfer learning (VGG-16) with heatmaps for interpretability in classification tasks	Multi-class	Pretrained model with explanations	Dataset diversity is limited; not generalized to real-world settings
Naseer et al. (2021)	Deep neural network model CNN	Uses aggressive data augmentation to overcome limited training data and improve generalization	Small datasets	Improved generalization	No use of explainability or advanced model optimization techniques
Bashkandi et al. (2022)	CNN optimized by a metaheuristic algorithm	Integrates Political Optimizer and Particle Swarm Optimization for fine-tuning CNN hyperparameters	Parameter sensitivity	Superior convergence	Increased algorithmic complexity may hinder real-time applications
Shawon et al. (2025)	CS-InceptionV3	Introduces class-weighted loss and XAI to mitigate data imbalance and support transparent decisions	Imbalanced dataset	Better recall for rare classes	May cause bias toward minority classes in high-class-count scenarios
Verma and Singh (2022)	Transfer learning using DenseNet201	Presents a comparative framework using different deep models to determine the most efficient design for tumor classification	Model optimization	Comprehensive design	Not deeply tailored to MRI-specific noise and structure challenges
Kumar et al. (2021)	ResNet-50 and global average pooling	Employs residual learning and global average pooling to reduce overfitting and model complexity	Overfitting	Lightweight and accurate	Interpretability and clinical correlation not addressed
Alhassan and Zainon (2021)	Swish-based RELU activation function-CNN	Introduces a novel activation function (Hard Swish) to improve CNN learning performance	Training instability	Improved training dynamics	Needs broader validation across datasets and clinical settings
Hosny et al. (2025)	Ensemble model (DenseNet121 + InceptionV3)	Combines multiple deep models with explainable AI to boost performance and transparency	Tumor diversity	High accuracy + trust	High computational cost and training time
Athisayamani et al. (2023)	ResNet-152	Uses a deep pretrained model (ResNet-152) followed by dimension reduction for better classification	High dimensionality	Good for low-resource setups	Not end-to-end trainable; relies on manual feature extraction post-CNN
Malla et al. (2023)	VGG-16 with fine-tuning	Streamlines architecture using GAP to eliminate fully connected layers, improving speed and generalization	Noisy input	Efficient + deployable	No focus on interpretability or attention mechanisms

## 3 Methods and materials

### 3.1 Dataset preparation

The Brain Tumor Dataset developed by Viradiya (2021) is a widely used collection of annotated medical images aimed at facilitating machine learning research in the domain of automated brain tumor classification. The dataset consists of 4,589 labeled MRI scan images, which are divided into two classes: Brain Tumor (2,513 images) and Healthy (2,076 images). Each image represents a magnetic resonance imaging (MRI) slice of the human brain. These images are organized into separate folders according to their respective classes, allowing for efficient supervised learning workflows. The dataset supports binary classification tasks, making it particularly suitable for deep learning techniques such as convolutional neural networks (CNNs). Although the dataset lacks pixel-level annotations for tumor segmentation and does not contain metadata such as patient information, acquisition parameters, or tumor types, it still serves as a valuable resource for initial experimentation and model prototyping in medical imaging tasks. Furthermore, the variability in image quality, brightness, and contrast presents a realistic challenge, thereby fostering the development of more robust and generalizable models.

Table 2 summarizes the key characteristics of the Brain Tumor Dataset utilized in this study, including the total number of MRI scans, tumor categories, imaging modality, and resolution details. It highlights the distribution of samples among glioma, meningioma, pituitary tumors, and healthy cases, which is critical for understanding class representation during model training.

**Table 2:** Key characteristics of the Brain Tumor Dataset.

Feature	Description
Source	Kaggle (Preet Viradiya)
Total number of images	4,589 images
Classes	2 (Brain Tumor, Healthy)
Image format	JPEG
Image type	MRI brain scans
Tumor images	2,513 images
Healthy images	2,076 images
Image annotation	Image-level labels only (no segmentation or bounding boxes)
Color format	Grayscale and color images
Clinical metadata	Not available
Intended task	Binary classification
Limitations	No pixel-level labels, lack of tumor type categorization, no clinical metadata

**Figure 1:**
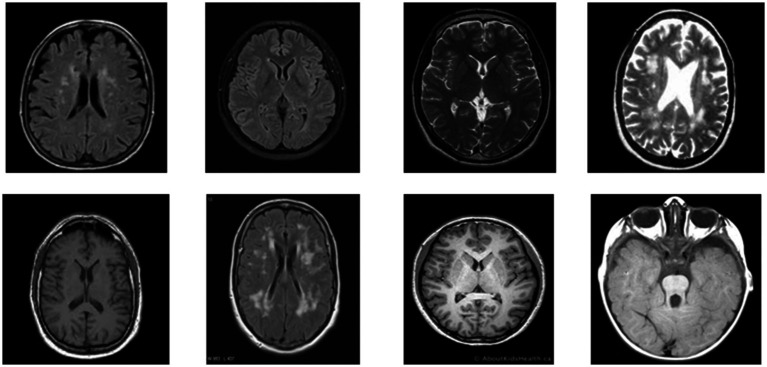
Visualization of healthy MRI scan.

**Figure 2:**
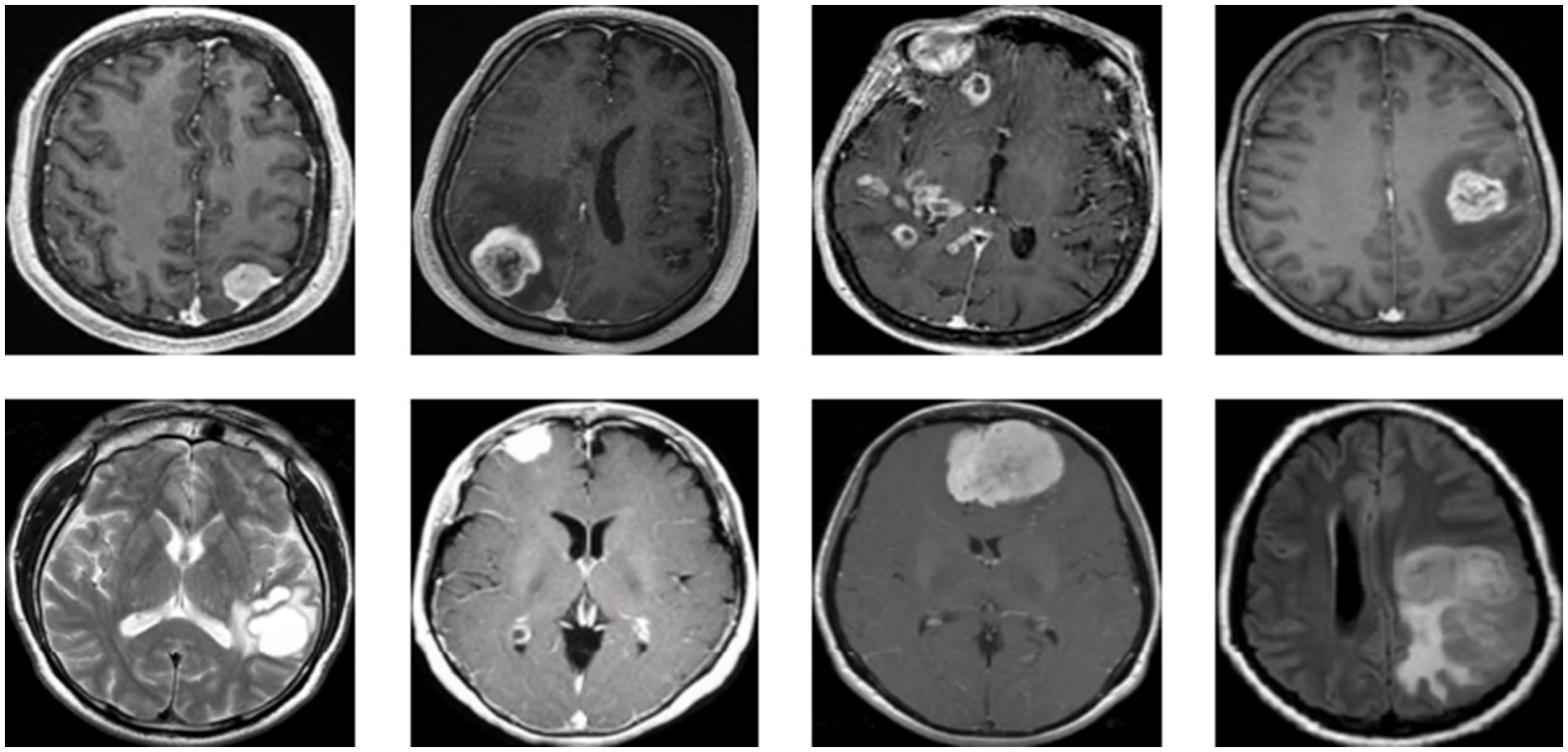
Visualization for tumor MRI scan.

**Figure 3:**
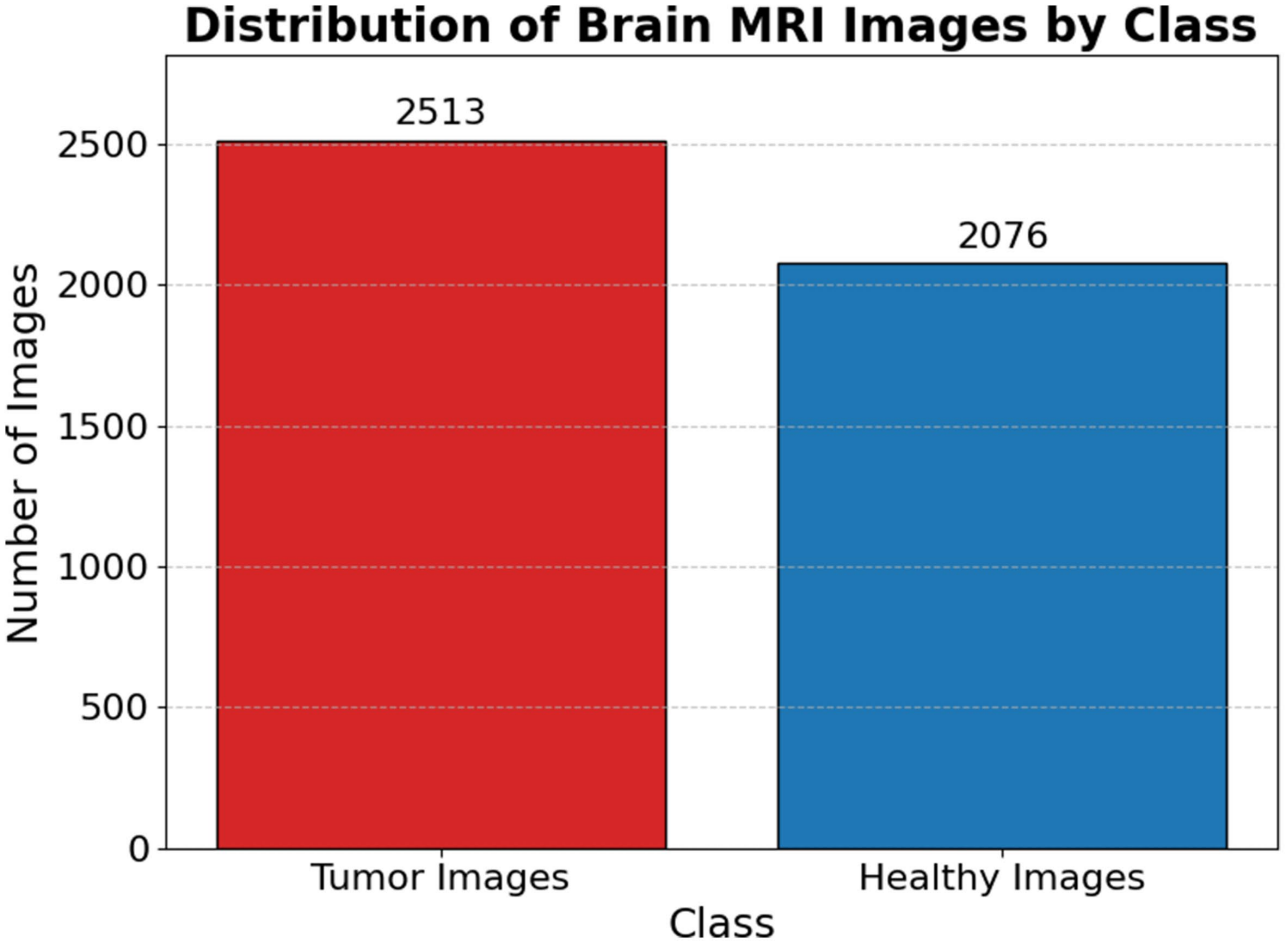
Distribution of brain MRI images scan by class.

Figure 1 presents a representative MRI scan of a healthy brain, illustrating normal anatomical structures without any abnormal growths. In contrast, Figure 2 visualizes an MRI scan with a visible brain tumor, showing abnormal intensity regions that are indicative of pathological tissues. Figure 3 illustrates the distribution of MRI images across the defined classes, revealing an inherent imbalance in the dataset, which was addressed using augmentation techniques and loss function adjustments to ensure model robustness and generalizability.

### 3.2 Data pre-processing strategy

To support robust model evaluation and ensure consistency in experimental design, this study was developed to automate the process of partitioning an image dataset into distinct subsets for training, validation, and testing. The function accepts parameters specifying the desired subset type, the proportion of data to allocate, the source directory containing the full dataset, and a mapping that indicates the number of images available in each class.

The methodology begins by verifying whether the target directory already exists; if it does not, it creates the necessary subdirectories for each class. A random number generator with a fixed seed of 42 is initialized to ensure that the selection of images remains consistent across multiple executions, promoting reproducibility. The function then retrieves the list of image files belonging to each class and determines the appropriate number of images to include in the current subset based on the specified split ratio. To prevent errors caused by excessively large split values, a conditional check ensures that the number of images selected does not exceed the total available.

**Figure 4:**
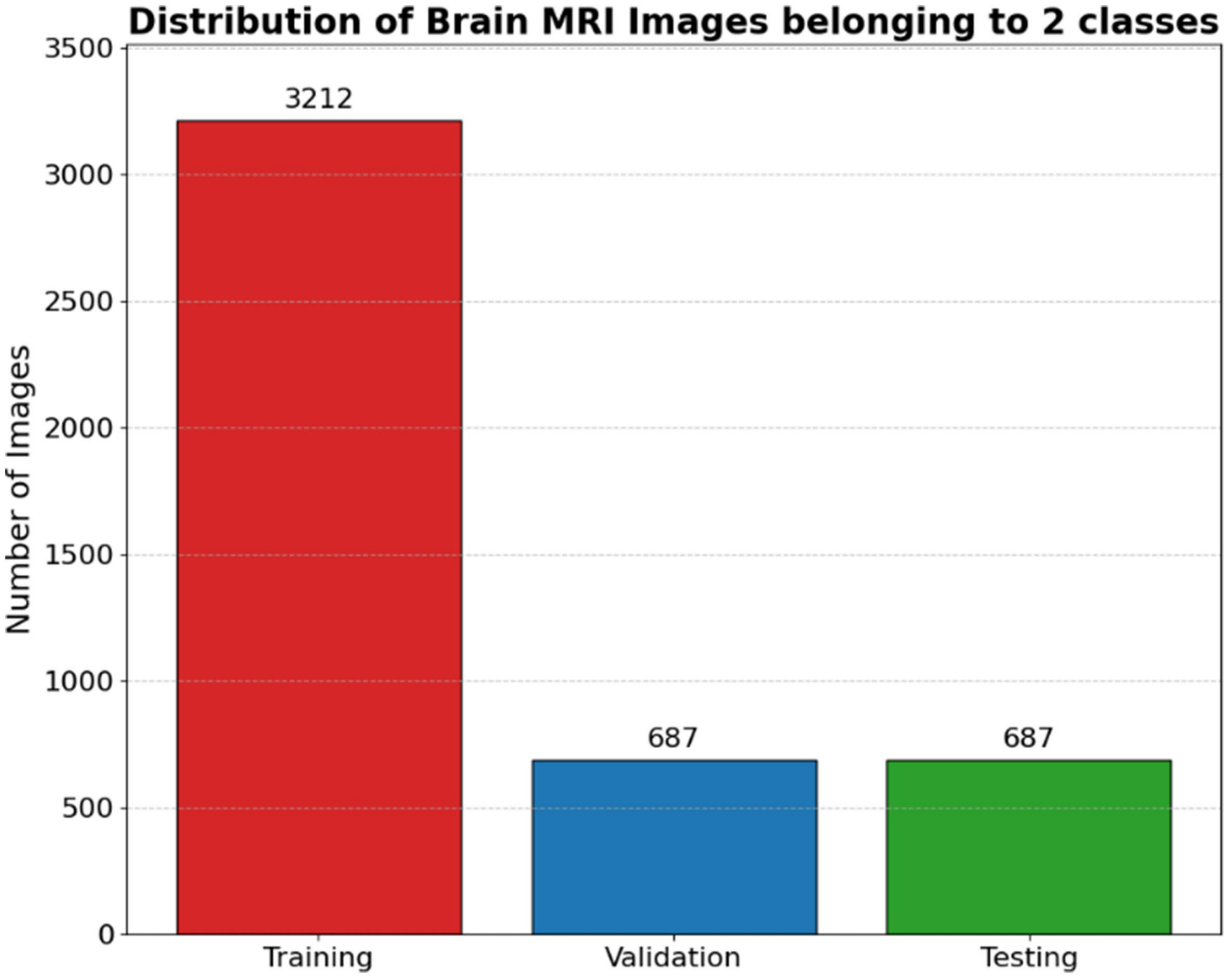
Distribution of brain MRI images belonging to 2 classes.

Once the appropriate number of images is randomly selected for each class, they are copied from the original dataset location to their corresponding destination folders within the target subset directory. This process is repeated for each class to maintain the original class distribution across all dataset splits. The function is subsequently invoked multiple times to create the training, validation, and testing sets using fixed proportions (e.g., 70, 15, and 15%, respectively) as illustrated in Figure 4.

This automated approach to dataset partitioning enhances both the scalability and reproducibility of the deep learning pipeline. It ensures a stratified and consistent dataset structure, which is crucial for developing reliable models, particularly in sensitive domains such as medical image classification.

In deep learning models, especially those dealing with image data, the quality and structure of the input pipeline are critical to achieving robust and generalizable performance. The create_image_generators function in TensorFlow/Keras is a high-level abstraction used to create these pipelines. It encapsulates both data preprocessing and data augmentation using the ImageDataGenerator class provides a continuous supply of mini-batches for training, validation, and testing.

Let each input image be represented by a tensor:


$$X\in R^{H\times W\times C}$$

where H and W are the height and width of the image, respectively, and C is the number of color channels (typically C=3 for RGB images).

Let the dataset consist of 𝑁 such samples:


$$D={\left\{(X_{i},y_{i})\right\}}_{i=1}^{N}$$

where $y_{i}\in {\left\{0,1\right\}}^{K}$ is a one-hot encoded vector denoting the class label for *K* categories.

The preprocessing function in deep learning—particularly in image classification tasks—refers to a transformation applied to raw input data before it is passed into a neural network. The primary goal is to ensure that input data is numerically stable, well-scaled, and statistically appropriate for the operations performed by the model’s layers. The preprocessing function applied in this context is a normalization technique defined mathematically as:


$$\text{preprocess}(x)=\frac{x}{127.5}-1,\;\;\;\;\forall_{x}\in [0,255]$$

This transformation operates on the raw pixel values of an image, where 𝑥 denotes the intensity of a given pixel. Typically, images are stored in an 8-bit format where pixel values lie in the range [0, 255]. The function first scales these values by dividing by 127.5, which brings them into the range [0, 2], and then shifts them by subtracting 1, resulting in a final normalized range of [−1, 1]. This type of normalization is referred to as linear normalization, since it preserves the relative proportions and distances between pixel values (i.e., it maintains the order and ratio of intensities).

The training datagen object created using ImageDataGenerator is a powerful tool for real-time data augmentation in image-based deep learning. It is designed to synthetically expand the training dataset by applying a series of random geometric transformations to each image. This helps prevent overfitting and enables the model to learn transformation-invariant features.

The data generator in this configuration applies a series of randomized image transformations to enrich the training dataset and improve model generalization. Each image is treated as a function 𝐼 (𝑥, 𝑦, 𝑐) where 𝑥, 𝑦 ∈ 𝑅 represent pixel coordinates and 𝑐 ∈ {1, 2, 3} denotes the RGB color channels. The augmented image $\hat{I}\;(x\prime ,y\prime ,c)$ is produced by applying a composition of geometric transformations followed by a pixel-level preprocessing function.

The full transformation pipeline can be summarized as:


$${\hat{I}}_{\text{final}}=\text{preprocess}\_\text{func}({T_{\text{flip}}}^{{}^{\circ}}{T_{\text{zoom}}}^{{}^{\circ}}{T_{\text{shear}}}^{{}^{\circ}}{T_{\text{shift}}}^{{}^{\circ}}T_{\text{rotate}}(I))$$

where 𝑇 represents the transformation functions applied sequentially.

Unlike the training data generator, which typically includes augmentation to artificially expand the dataset and improve generalization, these generators are configured solely with a preprocessing_function. This means that the only transformation applied to the validation and test images is a predefined pixel-level preprocessing step—commonly used to normalize or standardize image data. For example, the preprocessing function might scale pixel values to the range [0, 1], subtract the mean pixel value, or apply dataset-specific normalization. Applying the same preprocessing function to the training, validation, and test data ensures that all inputs are on the same scale and distribution, which is essential for a consistent model behavior and accurate evaluation. Importantly, no random changes such as rotation, flipping, or shifting are applied to the validation and test sets, as these datasets are meant to reflect real, unmodified data for unbiased performance measurement.

Table 3 outlines the augmentation strategies applied using the ImageDataGenerator function, including transformations such as rotation, zooming, shifting, and horizontal flipping, which introduced variation and improved the model’s ability to generalize across unseen MRI scans.

**Table 3:** ImageDataGenerator transformations.

Transformation	Description	Parameter value	Mathematical representation
Rotation	Rotates image around its center	rotation_range = 20	$\left[\begin{array}{c} x\prime \\ y\prime \end{array}]=R_{\theta }[\begin{array}{cc} x & c_{x}\\ y & c_{y} \end{array}]+[\begin{array}{c} c_{x}\\ c_{y} \end{array}\right]$ where $R_{\theta }$ is the rotation matrix
Width shift	Translates image horizontally	width_shift_range = 0.2	x′=x+Δx,Δx∈[−0.2W,+0.2W]
Height shift	Translates image vertically	height_shift_range = 0.2	y′=y+Δy,Δy∈[−0.2H,+0.2H]
Shearing	Slants image along horizontal direction	shear_range = 0.2	$\left[\begin{array}{c} x\prime \\ y\prime \end{array}]=[\begin{array}{cc} 1 & \lambda \\ 0 & 1 \end{array}][\begin{array}{c} x\\ y \end{array}],\lambda \in [-0.2,0.2\right]$
Zooming	Scales image in or out centered at image center	zoom_range = 0.2	$x\prime =z(x-c_{x})+c_{x},$ $y\prime =z(y-c_{y})+c_{y}$ z∈[0.8,1.2]
Horizontal flip	Flips image left-to-right with 50% probability	horizontal_flip = True	x′=W−x,y′=y
Fill mode	Fills empty pixels after transformation using nearest pixel	fill_mode = “nearest”	$\hat{I}\;(x\prime ,y\prime )=I(\text{nearest}(x\prime ,y\prime ))$
Preprocessing Func	Custom function for pixel value standardization	preprocess_func (custom)	${\hat{I}}_{\text{final}}(x\prime ,y\prime ,c)=$ preprocess_func ($\hat{I}\;(x\prime ,y\prime ,c)$)

### 3.3 Adapted methodology

This architecture integrates a pre-trained InceptionV3 convolutional (Rastogi et al., 2023) backbone with custom-designed squeeze-and-excitation (SE) attention mechanisms (Misra, 2024) and an edge-based feature extraction pathway using fixed Sobel filters, culminating in a model that is both semantically rich and texture-aware.

The model starts by accepting images of size 299 × 299 × 3, aligning with the native input dimension of InceptionV3, a state-of-the-art convolutional neural network known for its strong performance on large-scale image classification tasks like ImageNet. The image is fed into two parallel branches: one being the InceptionV3 base network, and the other a Sobel-based edge detection branch.

The edge detection branch is grounded in classical image processing theory, where Sobel filters are used to approximate the first-order image gradients in horizontal ($G_{x}$) and vertical ($G_{y}$) directions (Timothy, 2025). These are defined as:


$$G_{x}=I*S_{x},G_{y}=I*S_{y}$$

where ∗ denotes convolution, 𝐼 is the input image and $S_{x}$, $S_{y}$ are the horizontal and vertical Sobel kernels, respectively. The gradient magnitude, capturing edge strength, is computed using:


$$G=\sqrt{G_{x}^{2}}+G_{y}^{2}$$

This edge map is then passed through a shallow CNN to learn edge-level discriminative features, which are pooled via Global Average Pooling (GAP) to produce a compact edge feature vector. Concurrently, the input image is passed through InceptionV3, which extracts deep, hierarchical features through a cascade of convolutional blocks. This model, pre-trained on ImageNet, retains generalized visual knowledge that helps improve convergence and performance on downstream tasks. However, the last layers (from a defined index) are unfrozen for fine-tuning, allowing the network to adapt to the specific task.

To enhance the representational power of InceptionV3 outputs, a squeeze-and-excitation (SE) block is applied. The SE block adaptively recalibrates channel-wise feature responses by modeling interdependencies among feature channels (Misra, 2024). This is achieved in three steps:

- Squeeze: Apply GAP to reduce spatial dimensions, producing a channel descriptor $z\in R^{C}$.
- Excitation: Pass 𝑧 through a bottleneck of two fully connected layers with activations (ReLU and Sigmoid) to generate channel-wise weights $s\in R^{C}\;$.
- Reweight: Multiply the original feature maps by these learned weights to emphasize informative channels.

Mathematically, for input feature map $F\in R^{H\times W\times C}$, the attention-modulated feature is:


$${\hat{F}}_{c}=F_{c}\cdot s_{c}\;\text{for each channel}\;c=1,\ldots ,C$$

After applying SE attention and global pooling, the network obtains a deep semantic feature vector. This is concatenated with the edge-based feature vector, effectively merging low-level edge descriptors with high-level semantic abstractions:


$$f_{\text{combined}}=\text{Concat}(f_{\mathrm{cnn}},f_{\text{edge}})$$

This fused vector is then passed through a dense layer with 1,024 neurons and ReLU activation, followed by a Dropout layer to mitigate overfitting. The final classification is performed using a softmax output layer, which assigns probability scores across the num_classes defined in the training set.

The model is compiled using the Adam optimizer, which is well-suited for problems involving sparse gradients and noisy updates, with a fixed learning rate of $10^{-4}$. The loss function used is categorical cross-entropy, appropriate for multi-class classification. An optional ReduceLROnPlateau callback monitors the validation loss and reduces the learning rate if the performance stagnates, helping to escape local minima and promote convergence.

This hybrid design is intelligent and well-justified. The integration of edge detection complements the semantic encoding from InceptionV3 by introducing texture-level cues that deep networks sometimes overlook. The SE block provides adaptive feature recalibration, addressing the issue of uniform treatment across channels, which is a known limitation in CNNs. Furthermore, the fusion strategy—combining handcrafted edge cues with deep features—reflects an informed design principle reminiscent of early fusion in multimodal learning.

One notable strength is the trainability control: by freezing most of the InceptionV3 layers and fine-tuning only the top layers, the model maintains generalization while still adapting to new data. This significantly reduces the risk of overfitting and shortens training time. Algorithm 1, describing the hybrid model that combines InceptionV3, squeeze-and-excitation (SE) blocks, and edge detection for brain tumor classification.

**ALGORITHM 1:**
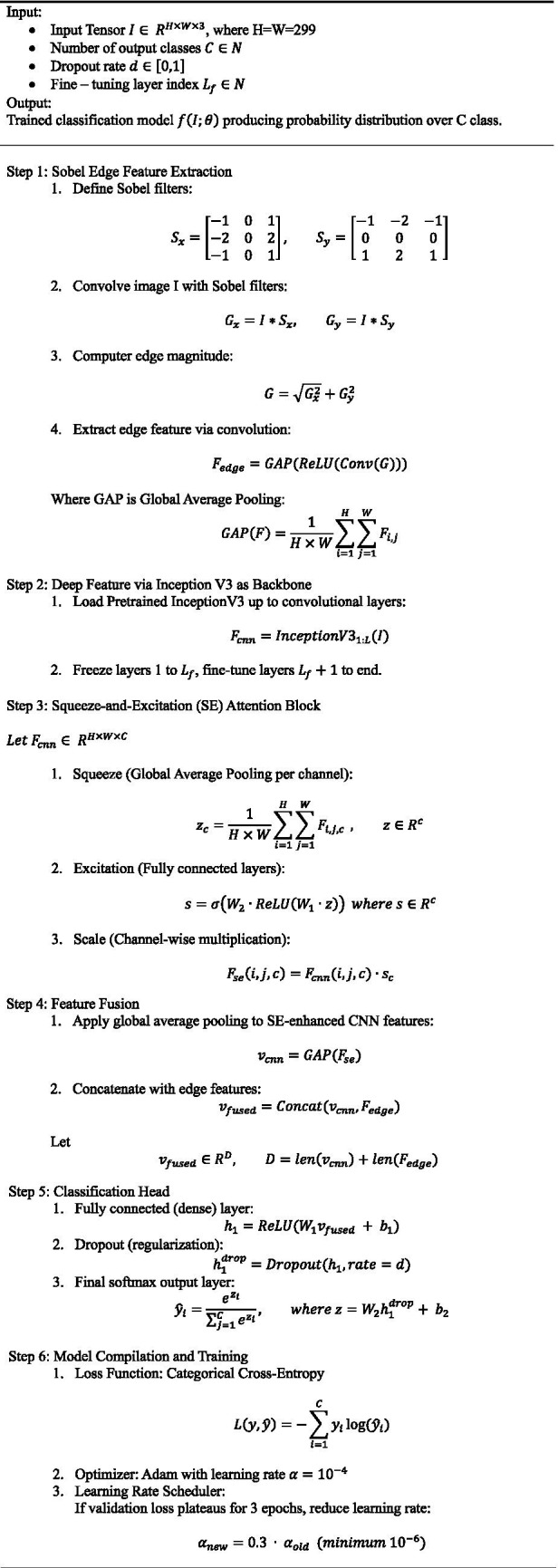
Algorithm step for the hybrid InceptionV3 + SE + edge detection model.

Table 4 highlights the key aspects of the proposed model architecture, detailing its core components including InceptionV3 backbone, SE blocks, edge detection integration, and classification layers. These elements collectively enhance accuracy, feature focus, and explainability in brain tumor prediction.

**Table 4:** Key aspect of the presented model architecture.

Aspect	Advantage
Edge-aware learning	Uses Sobel filters to extract explicit edge features, improving boundary awareness and spatial sensitivity—especially useful in texture- or shape-sensitive tasks (e.g., medical, remote sensing)
Dual-branch feature extraction	Combines traditional edge features with deep CNN features, leading to rich multi-scale representations. This improves robustness to appearance variations
Squeeze-and-excitation (SE) block	Enhances important features via channel-wise attention, increasing model’s focus on informative feature maps and reducing irrelevant noise.
Transfer learning efficiency	Leverages pretrained InceptionV3, reducing training time and requiring less labeled data while still achieving high accuracy
Fine-tuning flexibility	Enables selective fine-tuning starting at a configurable layer index (L_f), allowing a trade-off between computational efficiency and model adaptability
Compact & effective fusion	Uses global average pooling before fusion to reduce dimensionality and prevent overfitting, while concatenation retains complementary features

**Figure 5:**
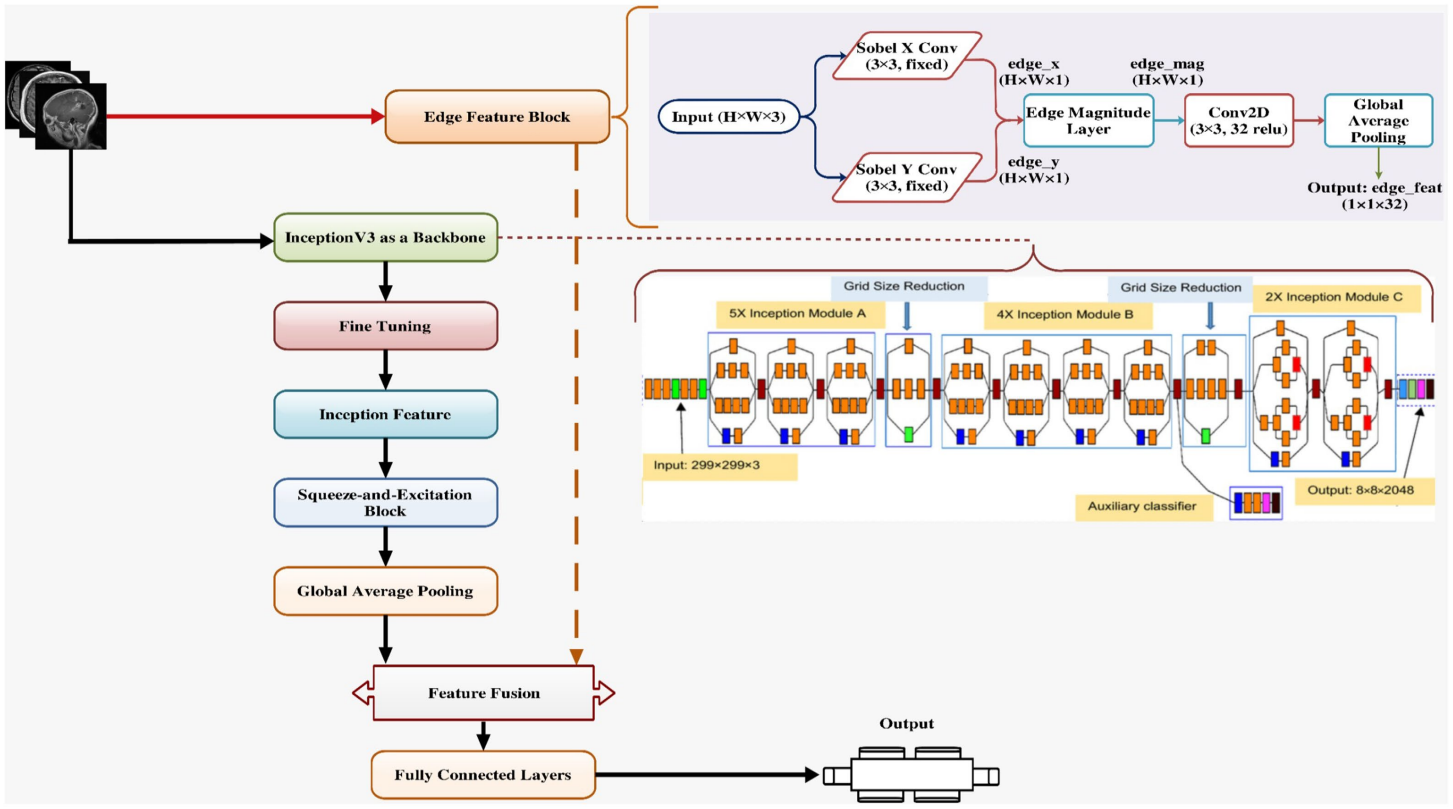
Proposed model architecture.

Figure 5 presents the proposed XAI-BT-EdgeNet architecture, combining InceptionV3 with squeeze-and-excitation blocks and edge detection to enhance feature representation. The next Table 5 is showing the hyperparameter list for the proposed model.

**Table 5:** List of hyperparameter.

Component	Hyperparameter name	Description
Input configuration	IMG_SIZE	Size of the input image to the model
SE (squeeze-excitation) Block	ratio	Reduction ratio used in channel excitation
Edge extraction block	filter_type	Type of edge detection filter (e.g., Sobel)
	trainable_filters	Whether the edge filters are trainable or fixed
	conv_filters	Number of filters in conv layer after edge detection
Base model (e.g., InceptionV3)	pretrained_weights	Source of pretrained weights (e.g., “imagenet”)
	include_top	Whether to include the original classifier layers
	trainable	Whether base model layers are trainable
	fine_tune_at	Layer index to start fine-tuning from
Classifier head	dense_units	Number of neurons in fully connected (Dense) layer
	dropout_rate	Fraction of input units to drop during training
	activation_output	Activation function for output layer
Optimizer settings	optimizer_type	Type of optimizer (e.g., Adam, SGD)
	learning_rate	Learning rate for the optimizer
Loss function	loss_function	Loss function for training (e.g., categorical cross-entropy)
Evaluation metrics	metrics	List of metrics to monitor during training and evaluation
Training configuration	epochs	Total number of training epochs
	steps_per_epoch	Number of steps per training epoch
	validation_steps	Number of validation steps per epoch
Callback—EarlyStopping	monitor	Metric to monitor for early stopping
	patience	Number of epochs with no improvement before stopping
	restore_best_weights	Whether to restore model weights from best epoch
Callback—ReduceLROnPlateau	monitor	Metric to monitor for reducing learning rate
	factor	Factor by which the learning rate will be reduced
	patience	Number of epochs to wait before reducing LR
	min_lr	Minimum learning rate value after reduction

## 4 Performance matrices

Performance metrics (Table 6) are quantitative measures used to evaluate the effectiveness of a model. These metrics provide insight into how well a model is performing on unseen data, guiding model selection, tuning, and deployment decisions.

**Table:** 

Symbol	Meaning
TP	True positive: Correctly predicted positive class
TN	True negative: Correctly predicted negative class
FP	False positive: Incorrectly predicted as positive
FN	False negative: Incorrectly predicted as negative
NN	Total number of samples
$y_{i}$	Actual label for sample *i*
$p_{i}$	Predicted probability for sample *i*
$w_{c}$	Class weight based on class frequency
$\mathrm{AU}C_{c}$	AUC score for class *c*
P, R	Precision and Recall (used in *F*1 formula)

**Table 6:** Performance matrices.

Metric	Definition	Formula	When to use	Interprets as
Accuracy	Accuracy evaluates the overall effectiveness of a classification model. It tells us how many of the total predictions were correct, without distinguishing between the types of errors	$\text{Accuracy}=\frac{\mathrm{TP}+\mathrm{TN}}{\mathrm{TP}+\mathrm{TN}+\mathrm{FP}+\mathrm{FN}}$	When classes are balanced	“How often did I get it right, regardless of class?”
Log Loss	Loss functions guide the training of classification models. Log Loss (used in binary classification) measures the difference between the actual label and the predicted probability. A lower loss indicates better predictions with more confidence	$\log\;\text{Loss}=-\frac{1}{N}\sum_{i=1}^{N}[y_{i}\cdot \log(p_{i})+(1-y_{i})\cdot \log(1-p_{i})]$	For probabilistic models, especially during training	“How far off was my prediction, especially if I was confident?”
Precision	Precision assesses the model’s ability to identify only the relevant (true positive) instances out of all the predicted positives. It is especially useful when false positives are costly	$\text{Precision}=\frac{\mathrm{TP}}{\mathrm{TP}+\mathrm{FP}}$	When false positives are more harmful	“Out of all predicted positives, how many were correct?”
Recall	Recall evaluates how well the model captures all the relevant positive instances from the actual data. It’s critical in scenarios where missing a positive case is highly undesirable	$\text{Recall}=\frac{\mathrm{TP}}{\mathrm{TP}+\mathrm{FN}}$	When false negatives are costly	“Out of actual positives, how many did I catch?”
*F*1 Score	The *F*1 Score provides a balance between precision and recall. It is the harmonic mean, which penalizes extreme values. A model with high *F*1 is both precise and has high recall	$F1\;\text{Score}=\frac{2\cdot (\text{Precision}\cdot \text{Recall})}{\text{Precision}+\text{Recall}}$	When needing trade-off between precision and recall	“How balanced is my model’s decision-making?”
Jaccard Score	This score evaluates similarity between the predicted and actual labels. It is particularly useful in multi-label and image segmentation tasks	$\text{Jaccard Score}=\frac{\mathrm{TP}}{\mathrm{TP}+\mathrm{FP}+\mathrm{FN}}$	For multi-label, segmentation, or set-based comparison	“How much do the predicted and actual labels overlap?”
Weighted AUC	AUC measures a classifier’s ability to distinguish between classes. In a multi-class problem, weighted average AUC considers each class’s importance based on its frequency in the dataset	$\text{Weighted}\;\mathrm{AUC}=\sum_{c=1}^{C}w_{c}\cdot \mathrm{AU}C_{c}$	For imbalanced multi-class problems	“How well does the model rank true classes across the board?”

Variable reference.

## 5 Results

**Figure 6:**
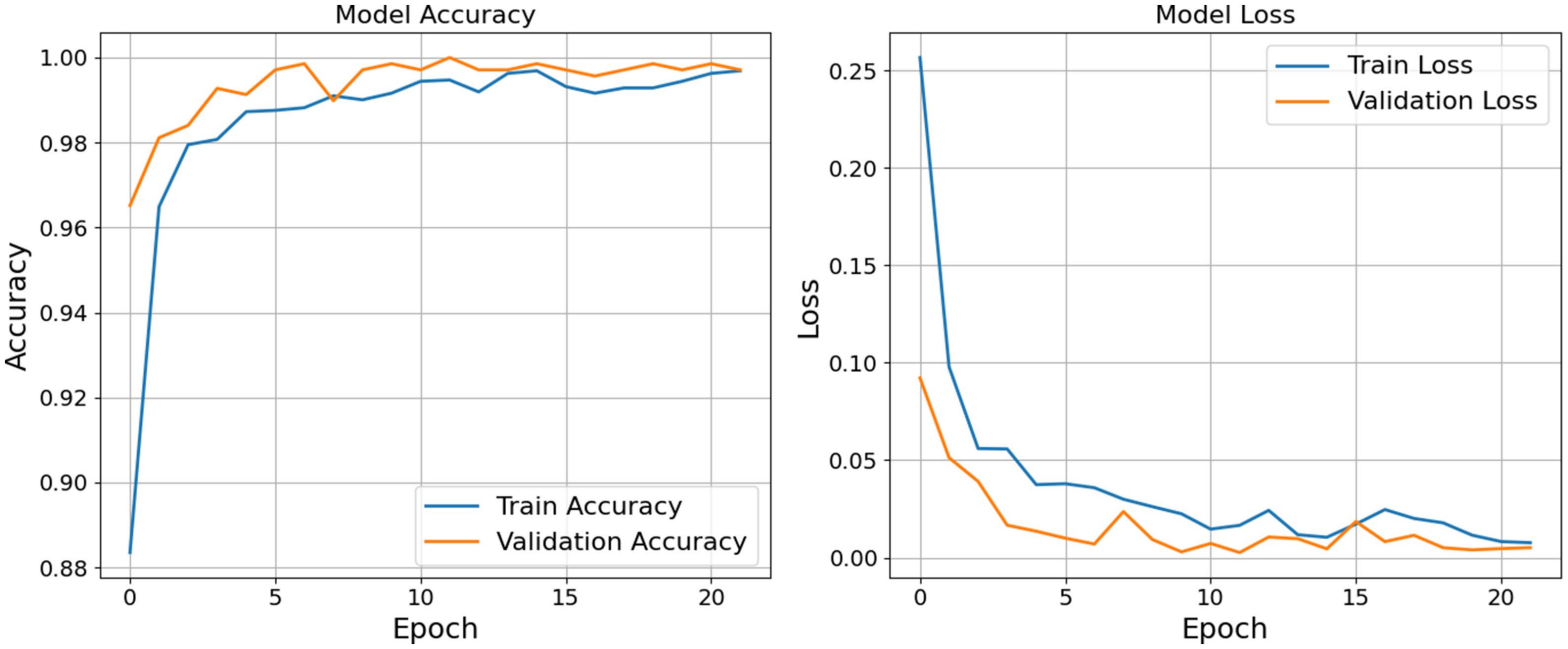
Accuracy and loss graph epoch by epoch.

As shown in the left subplot of Figure 6, the training accuracy exhibited a sharp increase during the initial epochs, rising from approximately 0.88 to over 0.98 within the first five epochs. This indicates that the model was able to rapidly learn meaningful patterns from the data. Beyond this point, accuracy improvements continued at a slower rate and plateaued near 0.9950, suggesting that the model reached a high level of predictive performance on the training data.

Similarly, the validation accuracy began at a relatively high baseline (around 0.9650) and approached 1.0000 within the first half of the training cycle. Notably, the validation accuracy remained consistently aligned with the training accuracy, demonstrating that the model maintained strong generalization capabilities across unseen data. The absence of significant divergence between the two curves implies that the model avoided overfitting, which is often a critical concern in deep learning applications.

The right subplot of Figure 6 displays the corresponding loss values. The training loss decreased substantially from an initial value above 0.25 to near zero, with a smooth and consistent downward trend. Minor oscillations observed after epoch 10 are typical and can be attributed to the stochastic nature of gradient-based optimization. More importantly, the validation loss also decreased consistently and remained lower than the training loss throughout the training period. This trend indicates not only that the model achieved accurate predictions but also that it was well-calibrated in terms of confidence in its outputs.

**Figure 7:**
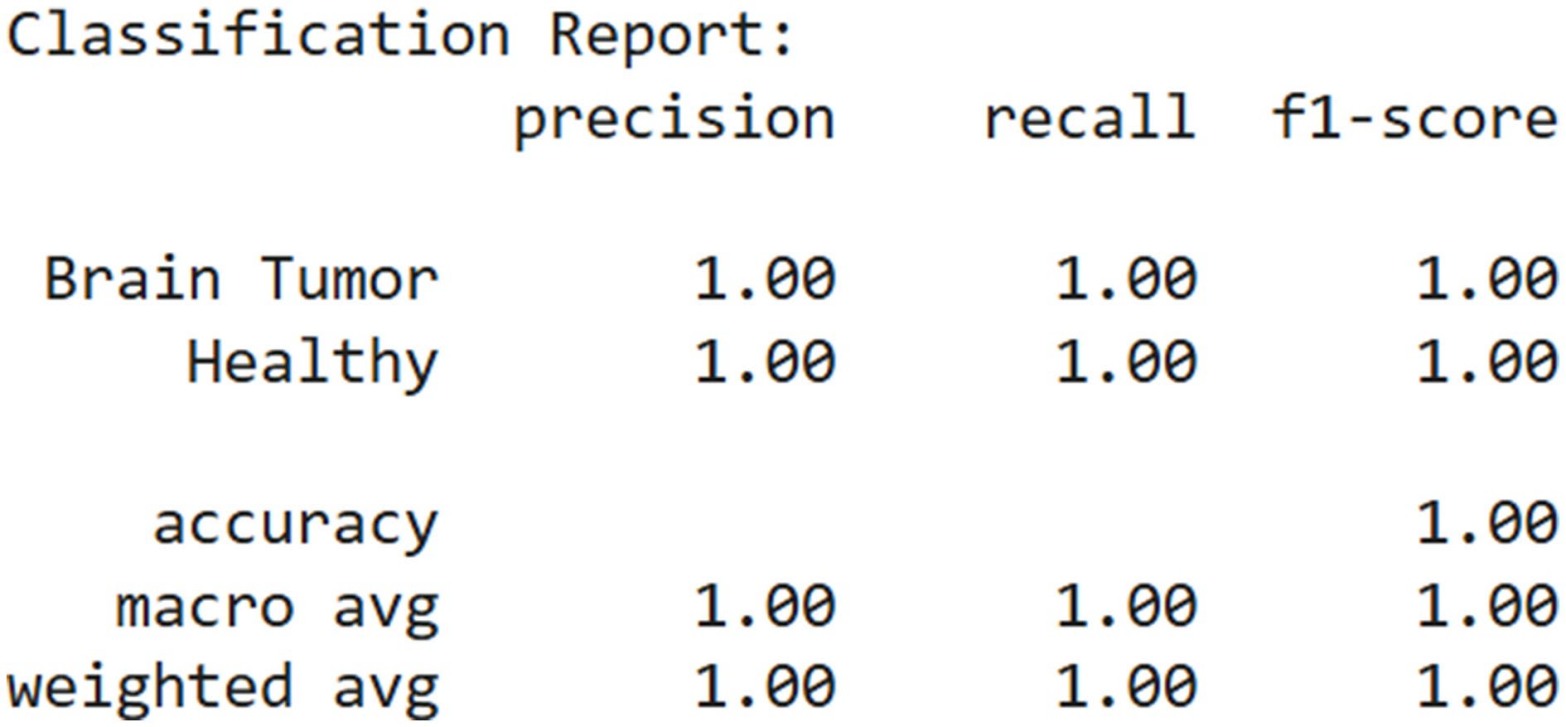
Classification report.

The combination of high accuracy and low, converging loss values for both training and validation sets suggests that the model was effectively optimized. The learning dynamics reflected in these plots indicate that the model was neither underfitting nor overfitting, and its generalization performance remained robust throughout the training process. Table 7 and Figure 7 shows the result for the proposed model.

**Table 7:** Result for accuracy and loss.

Matrices	Training	Validation	Testing
Accuracy	0.9958	0.9971	1.0000
Loss	0.0103	0.0051	0.0026

**Figure 8:**
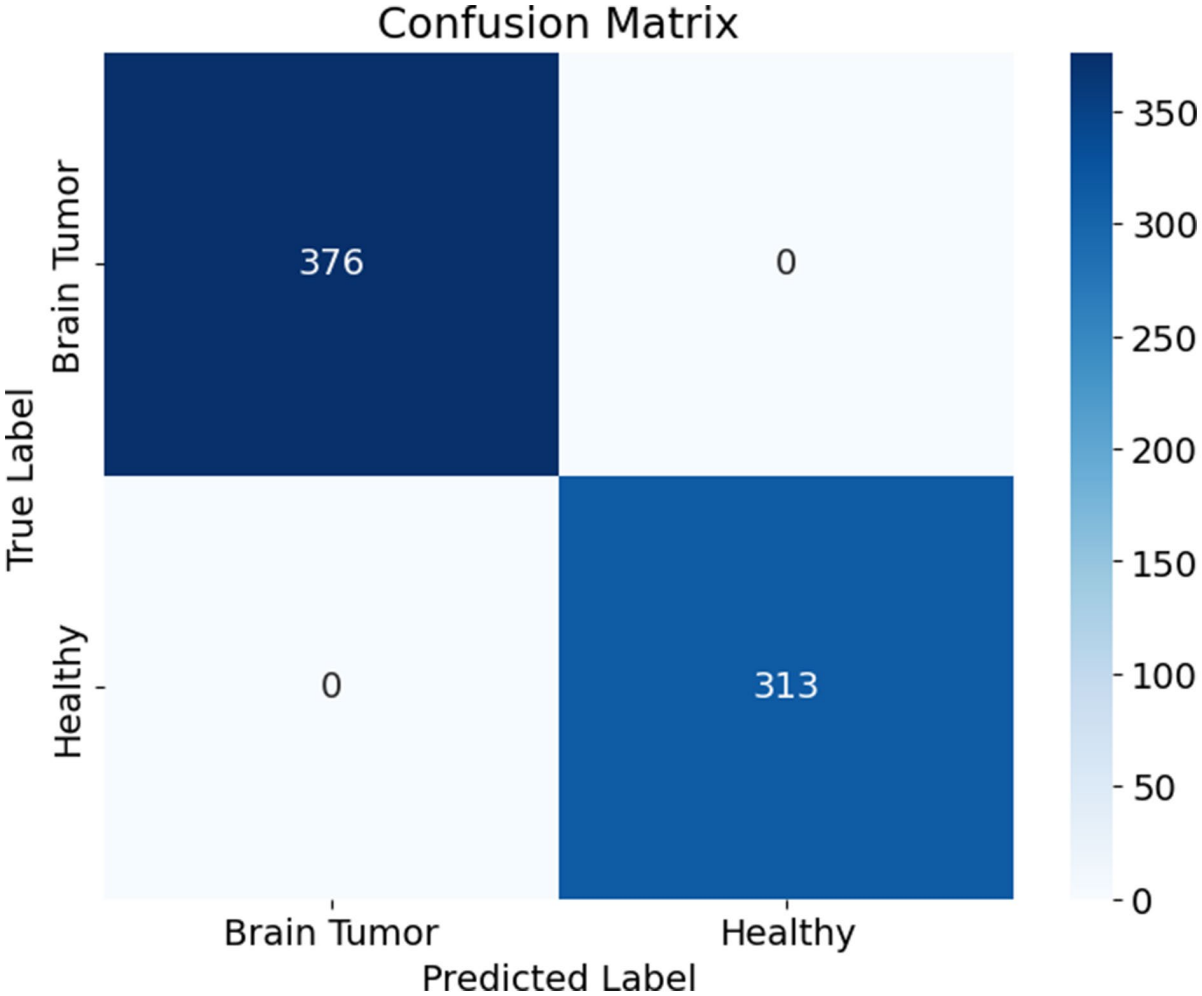
Confusion matrix.

The confusion matrix presented in Figure 8 illustrates the performance of a binary classification model designed to differentiate between brain tumor cases and healthy individuals. The matrix shows that the model correctly classified all 376 brain tumor cases as positive and all 313 healthy cases as negative, with no instances of misclassification. Specifically, the top-left cell indicates 376 true positives, meaning all patients with brain tumors were accurately identified. The bottom-right cell shows 313 true negatives, reflecting that all healthy individuals were correctly predicted as such. Notably, the matrix contains no false positives or false negatives, as indicated by the zero entries in the off-diagonal cells.

This outcome demonstrates that the model achieved perfect classification on the evaluated dataset, with 1.0000 accuracy, precision, recall, and specificity. The absence of any error implies that the model was highly effective at learning and distinguishing between the two classes. However, while such performance is impressive, it is essential to validate the model on an independent test set to confirm that it generalizes well to unseen data and is not merely overfitting the training or validation data.

**Figure 9:**
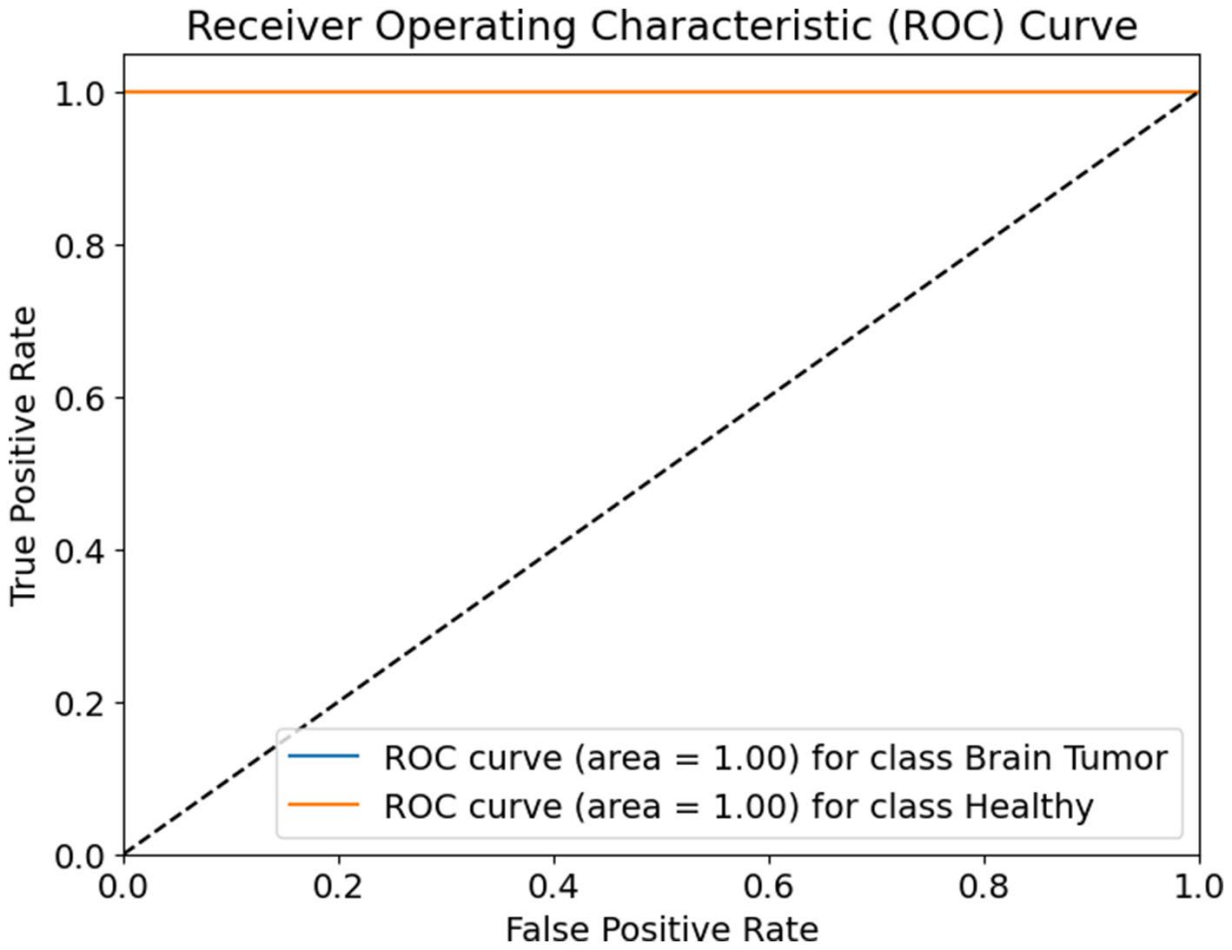
ROC curve.

The receiver operating characteristic (ROC) curve shown in Figure 9 evaluates the classification performance of a binary model distinguishing between two classes: brain tumor and healthy. The ROC curve plots the true positive rate (sensitivity) on the *Y*-axis against the false positive rate on the *X*-axis. The ideal ROC curve closely follows the top-left corner of the plot, indicating high sensitivity with low false positive rates.

In this case, the ROC curves for both classes—brain tumor and healthy—are represented as perfectly horizontal lines at the top of the graph. Both curves achieve an area under the curve (AUC) value of 1.00, which is the maximum possible score. This means that the model achieved perfect classification performance, correctly identifying all positive and negative instances for both classes without any confusion.

The diagonal dashed line represents a random classifier, where the model’s predictions are no better than random guessing. The fact that both ROC curves are well above this line confirms that the model’s performance is significantly better than chance. The curves reaching the top-left corner with zero false positives and 100% true positives for each class show that the model is both highly sensitive and highly specific, making it exceptionally reliable for the classification task at hand.

The displayed image shows a set of 10 brain scan samples, each labeled with both the ground truth (“True”) and the model’s classification result (“Pred”). In all five cases, the actual class is Brain Tumor, and the model has also correctly predicted Brain Tumor for each image. This visual representation provides qualitative evidence of the model’s effectiveness in correctly identifying brain tumor cases.

**Figure 10:**
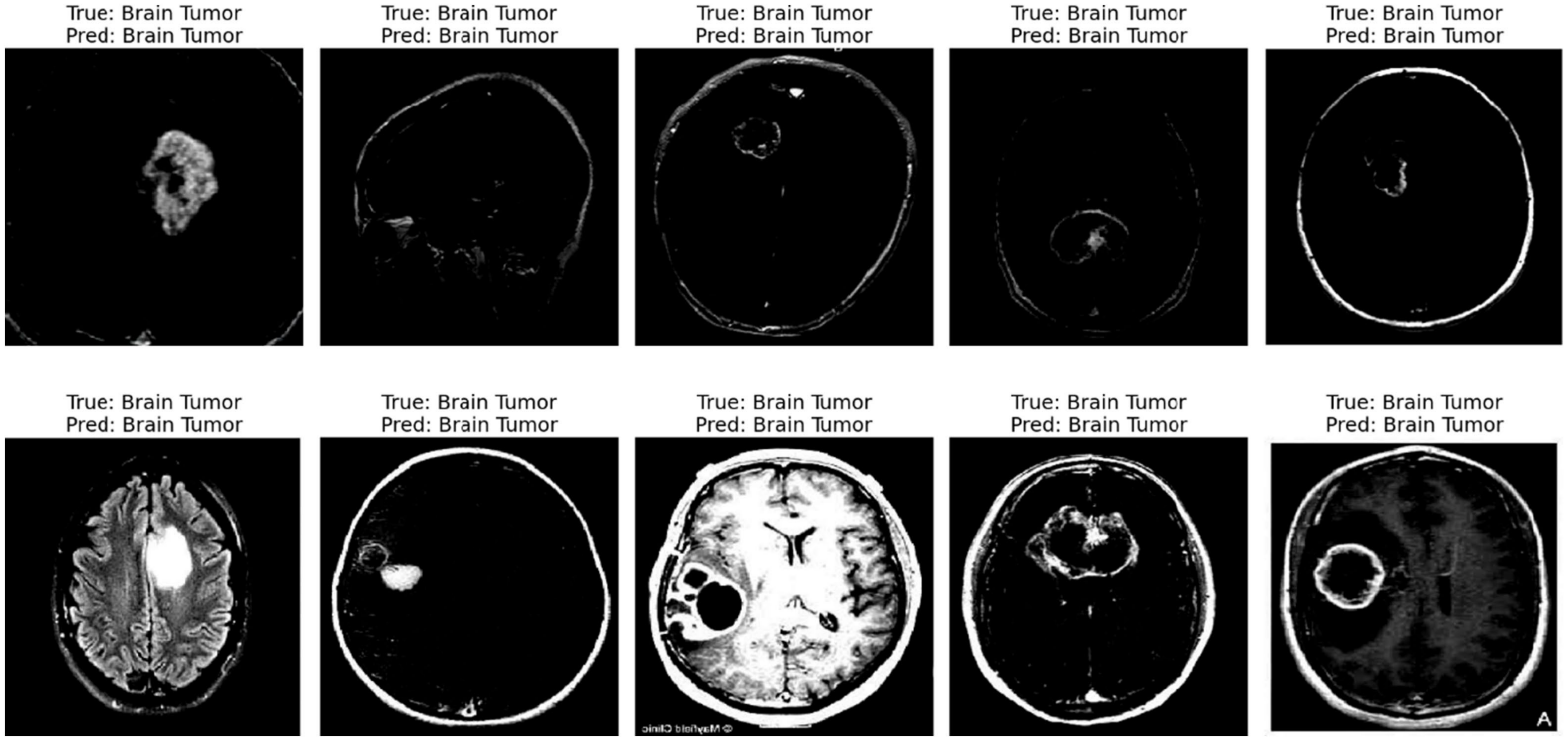
Predicted result.

Each image appears to be a medical imaging scan—likely from MRI modalities in Figure 10—processed in grayscale. The tumors are visible as brighter or differently textured regions in the brain scans, suggesting that the model was able to detect distinguishing visual features associated with tumorous growths. The consistency in correct classification across different tumor appearances indicates that the model has likely learned to generalize the underlying patterns of tumor presence effectively.

### 5.1 Explainable AI prediction

#### 5.1.1 LIME

**Figure 11:**
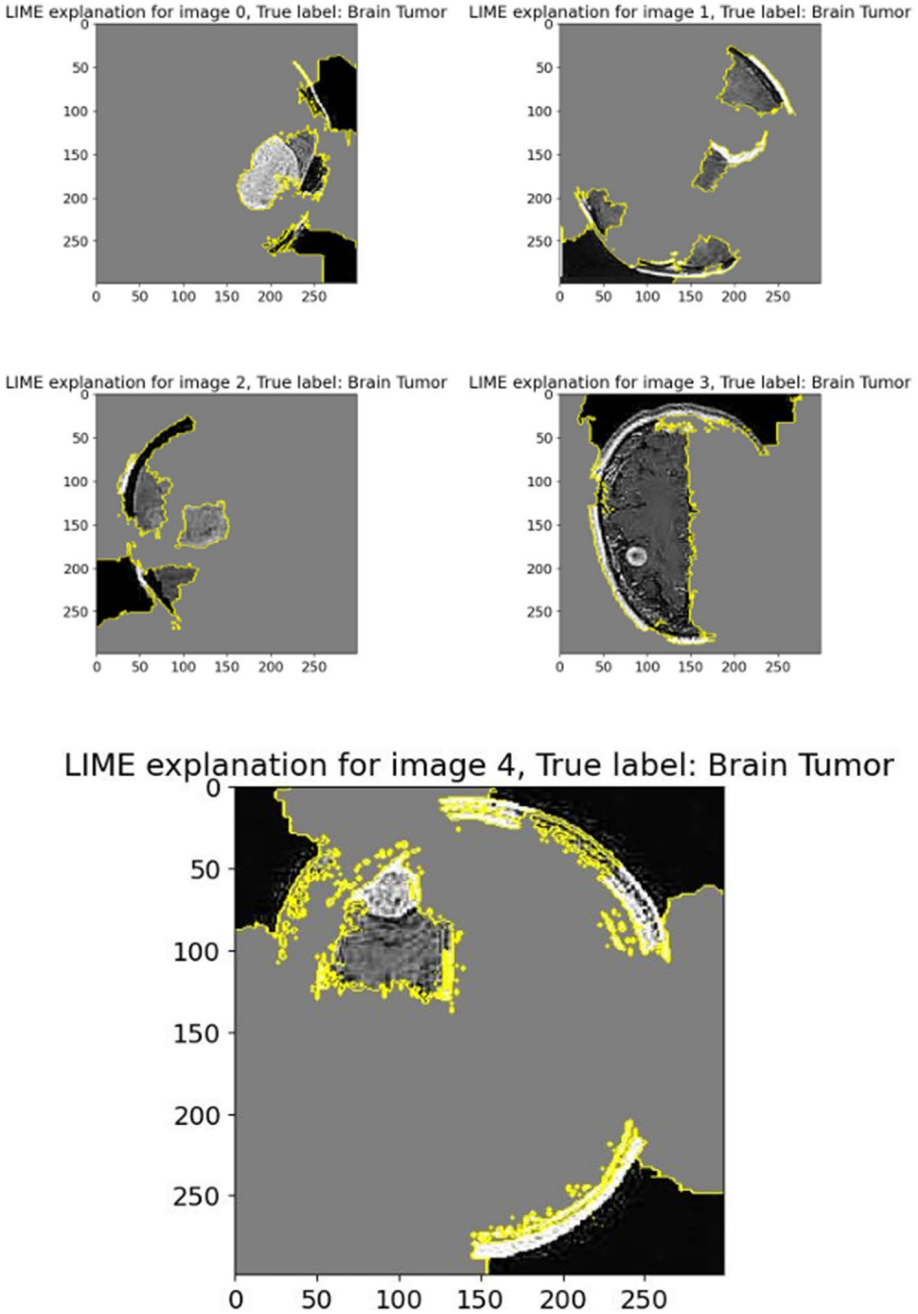
LIME explanation.

The displayed set of images (Figure 11) shows LIME (Local Interpretable Model-Agnostic Explanations) visualizations applied to five different brain scan samples, each labeled with the ground truth class “Brain Tumor.” LIME is a widely used interpretability technique that helps understand how a machine learning model arrives at a specific decision by highlighting which parts of the input image contribute most strongly to the classification (van der Velden et al., 2022; Mir and Pal, 2025).

In each of these grayscale brain scan images, different colored regions—primarily outlined in yellow—represent the superpixels or segmented regions that most influenced the model’s prediction toward the correct class (brain tumor). These areas are identified as being the most relevant in driving the model’s decision-making process. The gray background typically indicates less influential or neutral regions that had little or no effect on the final output.

For instance:

- In Image 0, a visible mass is located centrally and is surrounded by highlighted regions, showing that the model focused directly on the tumor-like structure.
- Image 1 shows segmentation in multiple dispersed areas, particularly near the upper and lower regions of the scan, suggesting the model has used both tumor features and possibly structural information from the surrounding brain anatomy.
- Images 2 and 3 illustrate segmentation in non-central regions, but still contain some tumor-dense sections within the highlighted zones. This implies that LIME identified both relevant tumor zones and adjacent tissues as contributory.
- In Image 4, the large highlighted patch precisely overlaps with a distinct tumor shape, indicating that the model heavily relied on this particular region to make its prediction.

These LIME explanations serve as a crucial tool for verifying that the model’s focus aligns with clinical expectations. For medical diagnosis tasks like brain tumor detection, it is essential to confirm that the classifier is basing its decisions on tumor-related patterns rather than irrelevant background features. LIME allows researchers and clinicians to inspect the model’s reasoning process, thereby increasing trust in its predictions and facilitating model transparency.

#### 5.1.2 GRAD-CAM

The image above presents Grad-CAM (Gradient-weighted Class Activation Mapping) visualizations applied to five MRI brain scan samples, all of which are correctly classified as showing a Brain Tumor. Grad-CAM is a powerful interpretability technique that helps understand which parts of the input image contribute most to a neural network’s decision, particularly in convolutional neural networks (CNNs) (Mir and Pal, 2025; Tsai and Lee, 2025).

In the Grad-CAM heatmaps:

- The red to yellow regions represent high model attention or importance—areas that had a strong influence on the model’s decision to classify the scan as showing a brain tumor.
- The blue to green regions represent areas of lesser importance.

The heatmap is superimposed on the original image to show where the model “looked” most carefully.

Let us break down the explanation per image:

- Image 0: The tumor is visible in the lower right area of the brain scan. The Grad-CAM heatmap highlights this same region in red, confirming that the model’s prediction is based on the actual tumor site.
- Image 1: The tumor appears slightly more complex and irregular in structure. The heat-map shows attention on the central part of the brain, especially around the bright white tumor region, suggesting the model used this area to identify the abnormality.
- Image 2: This scan is a side profile (sagittal view), making interpretation more complex. Despite the different orientation, the Grad-CAM heatmap still highlights the central mass with strong attention, indicating that the model adapted well and localized the tumor area accurately.
- Image 3: The tumor appears to occupy a deeper region of the brain. The model again focuses on this region, with the heatmap showing a dense red spot right on the tumor, reinforcing the reliability of the model’s detection.
- Image 4: The tumor here is located in the upper right quadrant of the scan. The heatmap aligns with this mass, highlighting it in red and confirming that the model’s prediction was influenced by the actual tumor.

**Figure 12:**
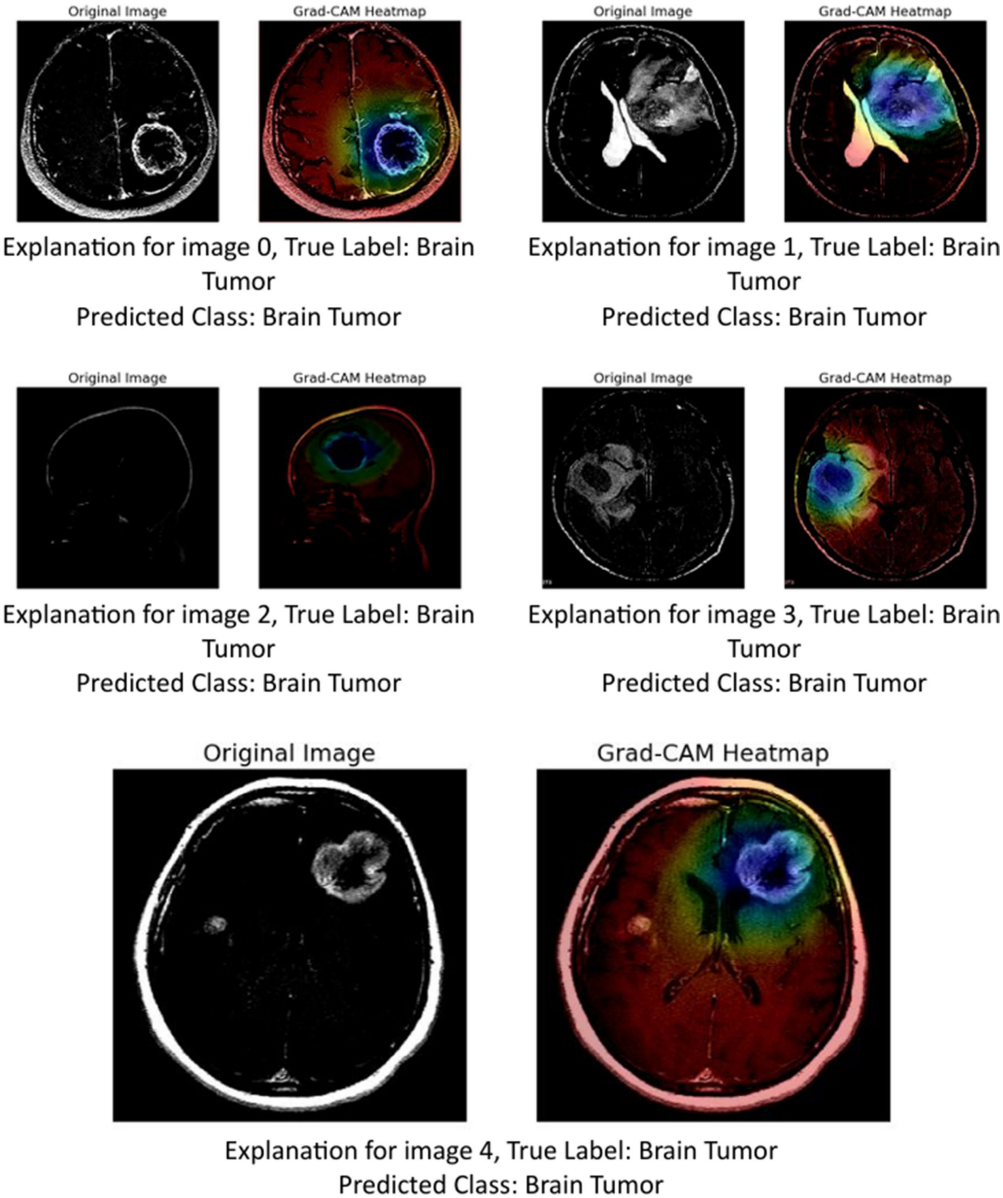
GRAD-CAM visualization.

These Grad-CAM visualizations demonstrate (Figure 12) that the model consistently focuses on the correct anatomical regions associated with brain tumors. The heatmaps provide visual confirmation that the CNN is not relying on irrelevant or misleading features, but rather, is making predictions based on medically significant areas. This enhances the interpretability and trustworthiness of the AI model in a sensitive application like medical diagnostics, where understanding the reasoning behind a decision is crucial for clinical validation.

#### 5.1.3 GRAD-CAM++

The provided image set presents a series of Grad-CAM++ visualizations applied to five brain MRI scans, who each diagnosed as having a brain tumor. These visualizations serve as an interpretability tool to understand the internal workings of deep learning models used in medical imaging. Grad-CAM++ (Gradient-weighted Class Activation Mapping++) is an enhancement over the original Grad-CAM technique, offering finer and more spatially precise localization of features that influence a model’s decision.

Grad-CAM++ improves upon Grad-CAM by considering higher-order derivatives of the output can coming the feature maps. This results in sharper and more reliable heatmaps, especially useful when multiple instances or small objects are present in an image (Chattopadhyay et al., 2022). In the context of medical imaging, such as MRI scans of brain tumors, Grad-CAM++ helps visualize which exact regions of the scan the model deems important for classification (Gao et al., 2023).

- Image 0:
  - Original image: Displays a clear structure of the brain, including the tumor area.
  - Grad-CAM++ heatmap: The central region, where the tumor is visibly located, shows a high activation (in red-yellow), indicating that the model strongly relied in this area to identify the tumor.
  - Observation: The tumor boundary and surrounding tissue are highlighted effectively, demonstrating good interpretability.
- Image 1:
  - Original image: Features a darker scan, but the tumor is discernible.
  - Grad-CAM++ heatmap: Activations are concentrated around the upper-mid portion of the brain, aligning well with the tumor location.
  - Observation: The model captures both the core of the tumor and some adjacent regions, suggesting sensitivity to contextual features.
- Image 2:
  - Original image: A clean scan with a bright abnormal region.
  - Grad-CAM++ heatmap: Strong focus in the central part, slightly diffused around the tumor area.
  - Observation: Grad-CAM++ shows focused activation, but also includes surrounding regions, possibly indicating model consideration of surrounding tissues.
- Image 3:
  - Original image: Exhibits distinct brain anatomy with a visible lesion.
  - Grad-CAM++ heatmap: High intensity near the center with tight boundary focus, particularly over the tumor.
  - Observation: Suggests that the model is highly accurate in pinpointing tumor locations, confirming the reliability of its internal decision logic.
- Image 4:
  - Original image: Displays a well-formed tumor in the lower right area.
  - Grad-CAM++ heatmap: The highest activation is located precisely over the tumor mass, with minimal distraction in other areas.
  - Observation: The result here shows a nearly perfect match between the medical region of interest and the model’s focus.

**Figure 13:**
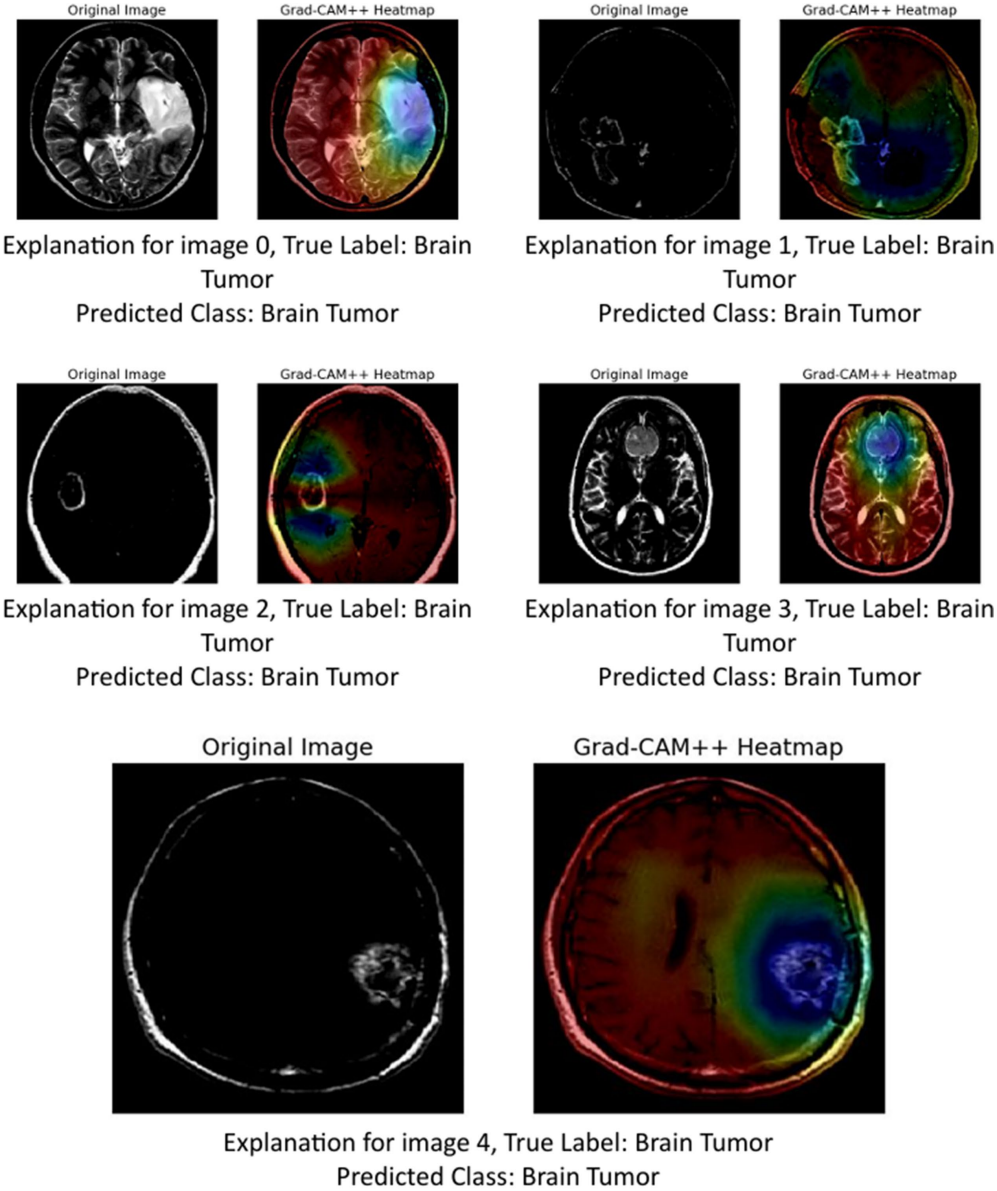
GRAD-CAM++ visualization.

These visualizations demonstrate (Figure 13) that Grad-CAM++ offers high-resolution, interpretable insights into CNN-based tumor detection models. It not only reveals the correct regions of interest (ROIs) within the MRI scans but also provides medical professionals with a visual validation of model predictions, which is essential for building trust in AI-assisted diagnostics. The consistent overlap between activated areas in the heatmaps and the actual tumor locations supports the use of Grad-CAM++ as a credible explainability method in medical imaging research.

#### 5.1.4 Vanilla Saliency

**Figure 14:**
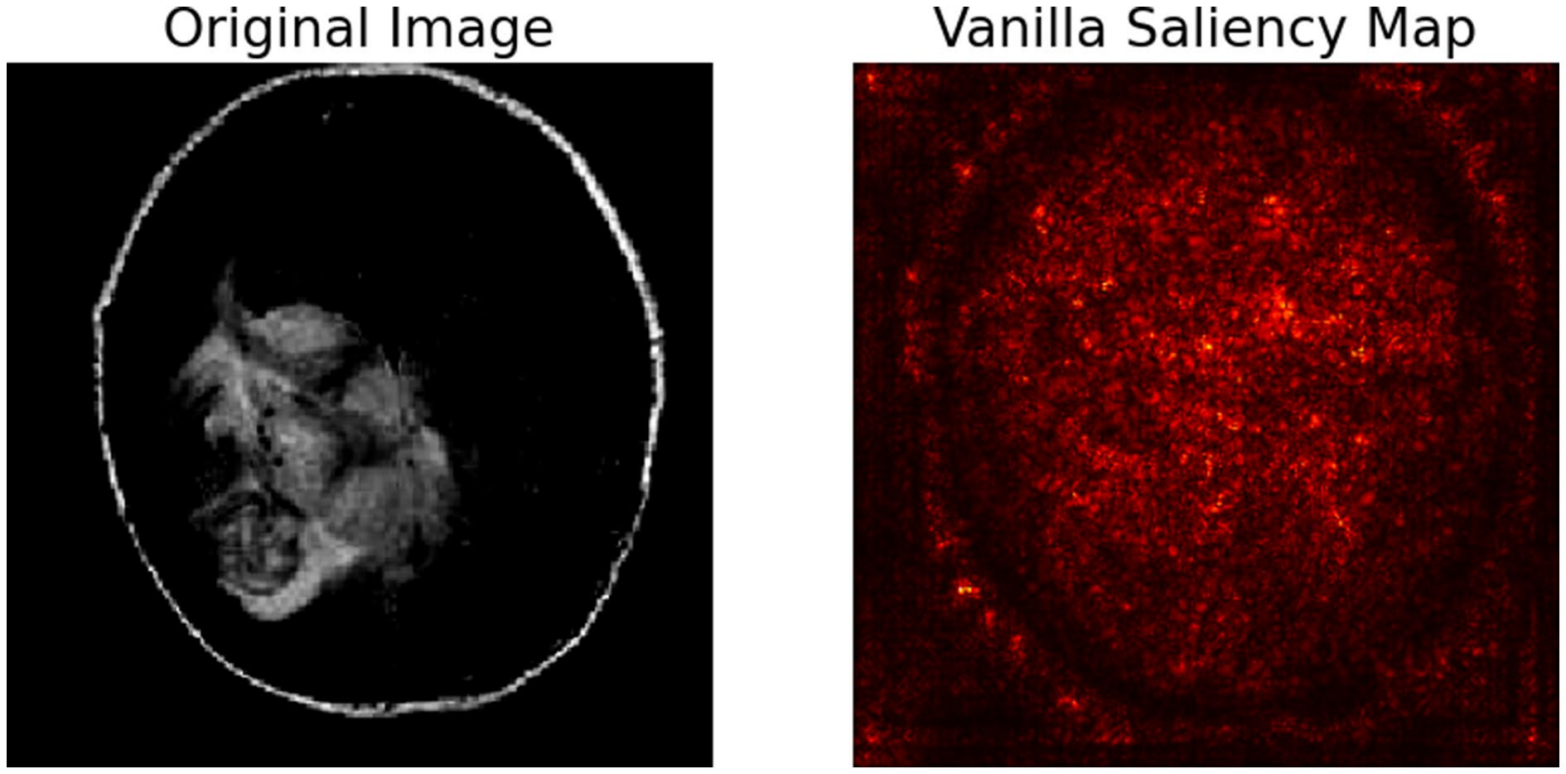
Vanilla Saliency map.

The image shows (Figure 14) a brain MRI (left) and its Vanilla Saliency Map (right), which highlights pixel regions influencing the model’s prediction. Vanilla Saliency computes gradients of the output to input pixels. Brighter areas indicate greater influence. However, the map appears noisy and lacks clear focus, making it less reliable for clinical use. While simple and fast, it’s often outperformed by advanced methods like Grad-CAM or LIME in interpretability and clarity.

## 6 Discussion

Table 8 presents a comparative overview of various deep learning architectures employed for multiclass and binary image classification tasks, highlighting their respective classification accuracies and the application of Explainable AI (XAI) methods. The primary goal of this comparison is to evaluate not only the predictive performance but also the interpretability of the models—a key requirement in high-stakes domains such as medical diagnostics. Among the surveyed models, the proposed XAI-BT-EdgeNet architecture demonstrates superior performance with an accuracy of 0.9958, surpassing all baseline and state-of-the-art methods listed. This is closely followed by ensemble and transfer learning-based approaches such as the Ensemble model (DenseNet121 + InceptionV3) (R18, 0.9902), ResNet-50 with global average pooling (R16, 0.9800), and VGG-16 with fine-tuning (R20, 0.9893).

**Table 8:** Comparative analysis for other literature model.

References	Model used	Classification	XAI method	Accuracy
Alzahrani (2023)	ConvMixer + Attention	Multiclass	—	0.9700
Rasheed et al. (2023)	CNN with Image Enhancement	Multiclass	—	0.9700
Imam and Alam (2023)	VGG-CNN	Multiclass	—	0.9600
AlTahhan et al. (2023)	Hybrid CNNs	Multiclass	—	0.9700
Özkaraca et al. (2023)	Dense CNN	Multiclass	—	0.9600
Peng and Liao (2023)	CNN-24 Layers	Multiclass	—	0.9400
Gómez-Guzmán et al. (2023)	InceptionV3	Multiclass	—	0.9700
Shanjida et al. (2022)	CNN-KNN	Multiclass	—	0.9500
Mercaldo et al. (2023)	Resnet50	Binary	GRAD-CAM	0.9900
Gaur et al. (2022)	CNN with dual-input	Multiclass	LIME, SHAP	0.8500
Ahmed et al. (2023)	VGG16	Binary	LRP	0.9700
Naseer et al. (2021)	Deep neural network model CNN	Multiclass	—	0.9881
Bashkandi et al. (2022)	CNN optimized by a metaheuristic algorithm	Multiclass	—	0.9709
Shawon et al. (2025)	CS-InceptionV3	Multiclass	—	0.9231
Verma and Singh (2022)	Transfer learning using DenseNet201	Multiclass	—	0.9822
Kumar et al. (2021)	ResNet-50 and global average pooling	Multiclass	—	0.9800
Alhassan and Zainon (2021)	Swish-based RELU activation function-CNN	Multiclass	—	0.9860
Hosny et al. (2025)	Ensemble model (DenseNet121 + InceptionV3)	Multiclass	GRAD-CAM	0.9902
Athisayamani et al. (2023)	ResNet-152	Multiclass	—	0.9885
Malla et al. (2023)	VGG-16 with fine-tuning	Multiclass	—	0.9893
Proposed model	XAI-BT-EdgeNet	Multiclass	LIME, GRAD-CAM, GRAD-CM++, VANILLA SALIENCY	0.9958

These results reaffirm the established trend that deep feature fusion and transfer learning significantly enhance classification performance. Traditional convolutional models such as VGG-CNN (R3), Modified CNN (R5), and CNN-24 Layers (R6) achieve competitive accuracies ranging from 0.9400 to 0.9700, indicating the reliability of CNN-based architectures even without extensive fine-tuning or hybrid designs. Notably, some hybrid models like AlexNet-KNN (R4) and CNN-KNN (R8) also achieve robust accuracies, suggesting that classical machine learning components, when integrated with deep features, can yield effective results. It is worth noting that several models such as ResNet50 (R9) and VGG16 (R11), were applied in binary classification tasks and achieved high accuracies (0.9900 and 0.9700, respectively), though the binary nature of their classification may inherently lead to better performance metrics compared to a more complex multiclass settings.

While most models focus solely on achieving high accuracy, only a limited subset incorporates XAI techniques are vital for transparency and trustworthiness. The proposed XAI-BT-EdgeNet is notable not only for its performance but also for its rich interpretability, employing a diverse suite of XAI methods, including LIME, GRAD-CAM, GRAD-CAM++, and Vanilla Saliency Maps. This extensive integration of explainability tools enables deeper insight into model decision-making and supports clinical validation.

Other models that incorporate XAI include CNN with dual-input (R10) using LIME and SHAP, VGG16 (R11) with Layer-wise Relevance Propagation (LRP), and ResNet50 (R9), and the Ensemble model (R18) utilizing GRAD-CAM. These methods provide varying levels of post-hoc interpretability, but are relatively limited in scope compared to the multi-method framework of the proposed model.

Despite the clear utility of XAI, its adoption remains limited across the surveyed literature. A significant proportion of high-performing models (e.g., R1–R8, R12–R17, R19–R20) do not report the use of any XAI methods. This presents a critical research gap, particularly in medical imaging, where model transparency can be as important as accuracy for clinical adoption.

Interestingly, R10, which integrates LIME and SHAP, reports a comparatively low accuracy (0.8500), suggesting a potential trade-off between model complexity, dual-input designs, and classification performance. However, this is not a generalizable trend, as demonstrated by the proposed model and R18, both of which achieve state-of-the-art accuracy while incorporating explainability. This indicates that with thoughtful model design, it is possible to strike a balance between performance and interpretability.

Statistical significance analysis is a way of determining whether the difference or improvement observed in your experimental results is real and meaningful, or whether it could have happened just by chance. For this analysis, study has done the *t*-test for Table 8.

Step 1—Prior models’ accuracies

- Number of models (*n*): 20
- Mean: 0.9634
- Standard deviation ≈ 0.0366

Step 2—Proposed model accuracy


$${\bar{x}}_{\text{proposed}}=0.9958$$

Step 3—One sample *t*-test formula


$$t=\frac{{\bar{x}}_{\text{proposed}}-\mu_{\text{previous}}}{s/\sqrt{n}}$$

$$t=\frac{0.9958-0.9634}{0.0366/\sqrt{20}}=\frac{0.0324}{0.00819}=3.96$$

Degrees of freedom = *n* − 1 = 19

Step 4—Significance (*p*-value)

For df = 19, a *t*-value ≈ 3.96 gives


p<0.001

To statistically assess performance improvement, a one-sample t-test was conducted comparing the proposed XAI-BT-EdgeNet (accuracy = 0.9958) with the accuracies of 20 previously reported models. The prior studies had a mean accuracy of 0.9634 ± 0.0366. The obtained *t*(19) = 3.96, *p* < 0.001, indicating that the improvement offered by XAI-BT-EdgeNet is statistically significant.

The XAI-BT-EdgeNet model was trained on Google Colab Pro+, leveraging an NVIDIA A100 GPU with 40 GB VRAM, 52 GB system RAM, and Python 3.10. The setup and performance metrics are summarized below (see Table 9).

**Table 9:** Setup requirement.

Hardware/Software	Specification	Notes
GPU	NVIDIA A100, 40 GB VRAM	High-performance GPU available via Colab Pro+
CPU	32-core virtual CPU	Provided by Colab environment for preprocessing
RAM	52 GB system RAM	Supports dataset loading and training
Python version	3.10	Compatible with PyTorch and supporting libraries
Deep learning framework	Tensorflow Library	Used for model implementation
Batch size	32	Optimized for GPU memory usage
Training time	~45–50 min per epoch	Total ~3–4 h for full dataset (4,589 MRI scans)
Inference time	~30–50 ms per MRI scan	Efficient for real-time clinical usage
Model parameters	~45 million	Dual-branch CNN with SE and XAI modules

The proposed brain tumor classification framework utilizes a dual-branch architecture that integrates InceptionV3 with an Edge Feature Block, aiming to improve tumor detection by combining high-level semantic features with low-level edge information. The following tables are a comprehensive examination of the framework’s strengths (see Table 10).

**Table 10:** Advantages of the proposed XAI-BT-EdgeNet framework.

Component/Block	Technical mechanism	Core advantage	Detailed benefit	Clinical relevance
Edge Feature Block	Applies fixed Sobel X and Y filters (3 × 3) to extract horizontal/vertical gradients; combines with edge magnitude layer and 2D conv (3 × 3, ReLU) followed by Global Average Pooling	Enhances edge sensitivity	Captures low-level structural features (e.g., tumor boundaries, shape discontinuities) that semantic models may miss	Useful for identifying hard-to-spot or irregular tumor boundaries; supports differential diagnosis
InceptionV3 Backbone	Multi-scale convolutions with parallel paths (1 × 1, 3 × 3, 5 × 5 filters); pretrained on ImageNet	Captures semantic richness	Learns high-level abstractions like texture, mass, and shape variations across tumor types	Effectively distinguishes among different tumor classes (e.g., glioma vs. meningioma)
Fine-Tuning of Backbone	Trains top layers on medical MRI data while retaining low-level features from ImageNet	Improves domain adaptation	Adjusts weights to align with medical imaging patterns while preserving general visual knowledge	Avoids overfitting and ensures adaptability to patient-specific brain scans
Squeeze-and-Excitation (SE) Block	Learns channel-wise attention via global pooling → bottleneck (FC) → sigmoid gating	Focuses on relevant channels	Dynamically enhances informative feature maps while suppressing noisy or irrelevant ones	Improves robustness in detecting subtle tumor patterns and handles variations across patients
Global Average Pooling (GAP)	Replaces flattening with spatial averaging across feature maps	Reduces parameters	Minimizes overfitting, increases efficiency, and retains global spatial information	Makes the model lightweight and applicable for clinical tools with limited hardware (e.g., mobile, edge devices)
Feature Fusion Layer	Concatenates or aggregates edge and semantic features post-GAP before feeding into classifier	Combines complementary features	Integrates fine-grained boundary cues with high-level contextual understanding	Increases classification robustness and precision; enables better handling of tumors with mixed textures
Modular Design for XAI	Clearly separated processing paths and interpretable transformations (e.g., Sobel, attention weights)	XAI-friendly	Facilitates application of interpretability tools like Grad-CAM, LIME on individual branches	Supports explainable outputs to aid clinicians in decision-making and trust-building
Auxiliary Classifier (in InceptionV3)	Mid-level output supervision through additional classifier	Reduces gradient vanishing in deep layers	Stabilizes training and improves generalization	Helps achieve more stable convergence on noisy medical data with limited samples

Despite its numerous advantages, the framework also presents several limitations outlined below.

- Computational overhead: Dual-branch networks with edge and semantic paths, plus SE blocks and feature fusion result in increased memory and computational costs.
- Fixed edge filters: The use of fixed Sobel kernels (non-trainable) in the edge block may limit adaptability to different imaging modalities or conditions (e.g., different MRI machines, noise levels).
- Redundancy in feature fusion: The semantic and edge branches may generate overlapping or correlated features, especially after global average pooling.
- Spatial misalignment during fusion: Due to separate processing pipelines, edge and semantic features may differ in spatial semantics or resolution.
- Risk of overfitting on small medical datasets: The deep and complex structure of the model increases its capacity, which, in the absence of large-scale labeled MRI datasets, can lead to overfitting.
- Domain mismatch in pretraining: The InceptionV3 model is pretrained on ImageNet, which contains natural RGB images, unlike grayscale MRI scans. While fine-tuning helps, the low-level features learned from non-medical images may not transfer well to brain MRIs, potentially limiting performance.

## 7 Conclusion

This study introduced XAI-BT-EdgeNet, an explainable edge-aware deep learning framework enhanced with squeeze-and-excitation modules for the accurate detection and classification of brain tumors from MRI images. By integrating both semantic and edge-level features through a dual-branch architecture, the model effectively captures crucial visual patterns associated with tumor regions. The inclusion of multiple explainability techniques—LIME, Grad-CAM, Grad-CAM++, and Vanilla Saliency—not only improves interpretability but also fosters greater clinical confidence in the system’s decision-making. Experimental results on a well-structured Brain Tumor Dataset demonstrate exceptional accuracy across training, validation, and testing phases, underscoring the robustness and generalization capabilities of the proposed model.

In addition to high performance, the framework addresses a critical gap in medical AI by balancing predictive strength with transparency, a requirement for real-world deployment in healthcare environments. The combination of edge detection, attention-based feature recalibration, and XAI integration provide a comprehensive and clinically relevant solution for brain tumor analysis. Future work may extend this approach to multi-class tumor grading, real-time diagnostics, and integration with radiologist feedback to further validate its practical utility.

## Data Availability

The original contributions presented in the study are included in the article/supplementary material, further inquiries can be directed to the corresponding author. The code used and analyzed in this study is openly available on GitHub at the following repository: https://github.com/Ani2014/XAI-BT-EdgeNet.git.
